# Culturally Sensitive Approaches in Psychosocial Interventions to Enhance Well-Being of Immigrant Adults Diagnosed with Breast Cancer: A Systematic Review

**DOI:** 10.3390/ijerph22030335

**Published:** 2025-02-25

**Authors:** Melba Sheila D’Souza, Juanita-Dawne Bacsu, Arsh Sharma, Ashwin Nairy

**Affiliations:** 1 Nursing and Population Health, Thompson Rivers University, Kamloops, BC V2C 0C8, Canada; 2 School of Nursing, Population Health and Aging Rural Research (PHARR) Centre, Thompson Rivers University, Kamloops, BC V2C 0C8, Canada; jbacsu@tru.ca; 3 Faculty of Science, The University of British Columbia, Vancouver, BC V6T 1Z1, Canada; asharm40@student.ubc.ca (A.S.); anairy@student.ubc.ca (A.N.)

**Keywords:** breast cancer, oncology, cultural care, psychosocial well-being, immigrants, patient, health, quality of life, systematic review

## Abstract

Objective: The objective is to synthesize the literature on culturally sensitive approaches in psychosocial interventions to enhance the well-being of immigrant adults diagnosed with breast cancer. Methods: We conducted a systematic review following the guidelines for Preferred Reporting Items for Systematic Reviews and Meta-Analysis (PRISMA) and reporting literature searches, and a multi-database search strategy of qualitative research studies and reports published in academic journals and grey literature within a 20-year duration. Results: We extracted data from twenty-two studies that met the inclusion criteria. Content analysis revealed experiences of cultural considerations in the care and psychosocial well-being of immigrants such as the development of culturally responsive care models; barriers and gaps in culturally responsive care in rural communities; patient information, education, and culturally responsive care; cultural stigma, and self-perception of the access, use, and role of healthcare providers, the impact of cancer and linguistically appropriate care; and challenges with psychosocial well-being and culturally responsive care. Conclusions: Concerns relating to psychosocial well-being of immigrant adults diagnosed with breast cancer are consistently described in the literature. Interventions exist to address psychosocial well-being; however, none have been developed or tested in immigrant adults diagnosed with breast cancer. Addressing the psychosocial well-being of immigrant adults will require the integration of culturally appropriate considerations in care to attitudes impacting patient care and reported outcomes.

## 1. Introduction

Breast cancer represents a significant public health concern, with an estimated 30,500 Canadian women being diagnosed, comprising 25% of all new cancer cases [1,2]. The projected direct costs of breast cancer are CAD 37 billion and CAD 143010 projected direct health systems costs per person [2]. The distribution of breast cancer cases by ethnicity reveals that, from 2006 to 2016, Canada, West Central Asian, and Middle Eastern populations accounted for 31% of cases, compared to 30.6% for African, 32.4% for South Asian, and 30.5% for East Asian individuals [3]. When it comes to breast cancer deaths during the same period and demographic breakdown, the percentages were 14.9% for West Central Asian and Middle Eastern, 11.5% for African, 9.1% for South Asian, and 20.8% for East Asian populations [3]. Despite recent developments in breast cancer screening, diagnosis, and treatment, there remains a significant lack of understanding regarding socioeconomic disparities and inequities in cancer outcomes. These global disparities are primarily attributed to unequal access to cancer care among immigrant populations affected by cancer.

Between 2016 and 2024, Canada witnessed the arrival of 3,049,277 non-permanent residents and 431,511 asylum persons and 2,617,766 permit holders and their family members [4,5]. The health of immigrants is influenced by education, employment, language, cultural differences, and differing beliefs about health [6]. The limited accessibility for immigrants can significantly impact their health, leading to critical events, such as missing cancer screenings due to language barriers [6]. Language and health literacy, beyond being a communication tool, plays a crucial role for immigrant women, especially those with English as a second language and cultural and health belief barriers such as cancer-related stigma and that concerning help-seeking attitudes in conveying their healthcare and social challenges [7]. Healthcare providers recognize the cultural stigma associated with mental health issues and help-seeking behaviours among immigrant clients [8]. Global issues for immigrant women can vary due to diverse understandings of diseases, societal stigmas, and personal, social, cultural, and religious beliefs and values.

The previous literature demonstrates that income and education inequalities and barriers to culturally appropriate care have been linked to increased health inequities [9]. A language barrier poses a potential threat to informed communication and decision-making processes. It thus affects the extent and quality of information, control negotiation, and establishing a trusting professional relationship with healthcare providers [10]. Breast cancer programs require culturally competent communication, language and culture [11]. A lack of knowledge, isolation, poverty, and lower health literacy can hinder women diagnosed with and treated for breast cancer from seeking necessary healthcare support and resources, which affects their psychological and emotional well-being [12]. Participants exhibit positive attitudes yet inadequate knowledge and experience regarding cancer screening [13].

In the global context of the psychosocial well-being of immigrant adults, the integration of psychosocial care outcomes and cultural care throughout the cancer care continuum is important as it can impact work productivity and the overall quality of life for individuals affected by cancer. This systematic review was conducted to provide a perspective, transfer knowledge, identify best practices, and address research gaps. This review aimed to learn from global experiences to inform care practices and discuss how findings from different healthcare systems might be adapted to fit healthcare in Canada. This review will address potential limitations, such as differences in population demographics and services in healthcare. To our knowledge, this contextual systematic review is the first study to examine culturally sensitive approaches in psychosocial interventions to enhance the well-being of immigrant adults diagnosed with breast cancer in Canada.

### Aim and Purpose

The primary purpose of this systematic review is to synthesize the published literature on culturally sensitive approaches to enhancing the well-being of immigrant adults diagnosed with breast cancer. The secondary purpose is to identify interventions to address the psychosocial well-being of immigrant adults with breast cancer. Identifying knowledge gaps and synthesizing research in this area will provide practitioners and researchers with insight into the implications of the psychosocial well-being of immigrant adults with breast cancer and indicate areas where future research and interventions are necessary.

## 2. Materials and Methods

### 2.1. Design and Methods

We followed the Preferred Reporting Items for Systematic Reviews and Meta-Analysis (Supplementary File S5 PRISMA 2020 Checklist) systematic review guidelines as well as the PRISMA guidelines for reporting literature searches [14]. This is an appropriate rigorous methodological approach to mapping the core concepts related to the scope and breadth of the literature on the psychosocial well-being of immigrant adults with breast cancer; this Joanna Briggs Institute (JBI) methodology for qualitative systematic review [15] is suitable as the purpose is not to conclude on but to scope the nature of the literature and provide directions for future work. The qualitative review included peer-reviewed articles published between January 2001 and December 2021 to review the perspectives and experiences of culturally sensitive approaches to enhancing the well-being of immigrant adults. The inclusion criteria for the year of publication from January 2001 to December 2021 in the systematic review involves two decades during which advancements in psychosocial interventions have occurred and reflect ongoing methodologies, cultural considerations, and healthcare policies relevant to immigrant populations with breast cancer. This review helped to recognize the foundational concepts, compare experiences over time, and identify challenges in culturally sensitive approaches to enhancing the well-being of immigrant adults diagnosed with breast cancer.

### 2.2. Inclusion Criteria

We included peer-reviewed articles published in English and considered studies that included immigrant adult women who were at least 18 years old and had completed their breast cancer screening, diagnosis and treatment.

### 2.3. Exclusion Criteria

We excluded interventions outside the oncology setting, non-immigrant adults, advanced diagnoses of breast cancer, and patients who were then undergoing active treatment, palliative care, or end-of-life care.

### 2.4. Types of Studies

This systematic review considered studies that focused on qualitative methods, including, but not limited to, designs such as phenomenology, grounded theory, ethnography, action research, and feminist research to provide descriptions of people’s experiences of how culture and environment influence behaviours within their context. The qualitative review included articles published between January 2001 and December 2021 to review the trends and issues in culturally sensitive approaches to enhancing the well-being of immigrant adults.

### 2.5. Search Strategy

Following the development of our a priori protocol, we worked with a librarian and designed a comprehensive search of relevant databases to identify literature referring to the psychosocial well-being of immigrant adults diagnosed with breast cancer. We searched eight databases from 2001 to 2021—BCS PubMed/Ovid/MEDLINE(R)/CINHAL, Embase, PsycINFO, Web of Science, and Google Scholar—and systematically searched by using prespecified Medical Subject Heading (MeSH) terms and keywords. The precise search strategy, including all identified keywords and index terms, was adapted for each included information source in the full review (Supplementary Files S1 to S5). We used secondary search strategies to identify additional relevant studies by scanning the reference lists of relevant papers identified at the full-text screening stage.

### 2.6. Study Selection

We found 22 articles (see Table S1) from the year 2004 (1), 2009 (2), 2011 (1), 2012 (1), 2013 (4), 2014 (1), 2015 (1), 2016 (4), 2017 (1), 2018 (2), 2019 (1), 2020 (2), and 2021 (1). Major waves of immigration to Western countries occurred later in the 20th century, making immigrant health issues less prominent in earlier decades. Cancer research concentrates on medical treatments and survival rates rather than psychosocial well-being. Research involving immigrant populations was challenging due to language differences, limiting study designs and participant recruitment. The concept of cultural competence evolved gradually, gaining more attention in the late 20th and early 21st centuries.

All identified results were stored and loaded into the reference management software EndNote X20 (The EndNote Team, 2013, Clarivate, Philadelphia, PA, USA https://endnote.com/, accessed on 21 December 2020), and duplicates were removed. EndNote X20 is a reference management software developed by Clarivate Analytics. This version of EndNote was used for managing references in a systematic review. EndNote X20 allowed us to create, organize, and search reference libraries efficiently. EndNote X20 was used as an automation tool to provide access to online bibliographic databases, facilitating the retrieval and import of references from various sources. The software supported collaboration among the researchers, which is useful for systematic reviews involving multiple team members.

The two independent reviewers screened the article titles and abstracts for assessment against the inclusion criteria to be identified as relevant, not relevant, and maybe relevant. The authors screened all the articles to confirm their eligibility and identified potentially relevant articles. To ensure the validity of the selection criteria, in the EndNote library, the keyword searches of the article titles were deemed irrelevant for the reapplication of the selection criteria. All relevant studies were retrieved in full text, and their citation details were independently reviewed against the selection criteria to confirm their inclusion (Table S1). Any disagreements between the reviewers at each stage of the study selection process were resolved through discussions [16].

Assessment of bias and quality was completed for all the included studies (Table S1). The JBI Critical Appraisal Checklist for Qualitative Research (JBI CAC) was used for all the qualitative studies. The JBI CAC contained 10 items, each scored as Yes, No, and Unclear, and the scoring for each article was as follows: 7–10, High Impact; 4–6, Medium Impact; and 1–3, Low Impact. Q1 = Is there congruity between the stated philosophical perspective and the research methodology? Q2 = Is there congruity between the research methodology and the research question or objectives? Q3 = Is there congruity between the research methodology and the methods used to collect data? Q4 = Is there congruity between the research methodology and the representation and analysis of data? Q5 = Were those delivering treatment blind to treatment assignment? Q6 = Is there a statement locating the researcher culturally or theoretically? Q7 = Is the influence of the researcher on the research, and vice versa, addressed? Q8 = Are participants, and their voices, adequately represented? Q9 = Is the research ethical according to current criteria or, for recent studies, is there evidence of ethical approval by an appropriate body? Q10 = Do the conclusions drawn in the research report flow from the analysis, or interpretation, of the data?

**Figure 1:**
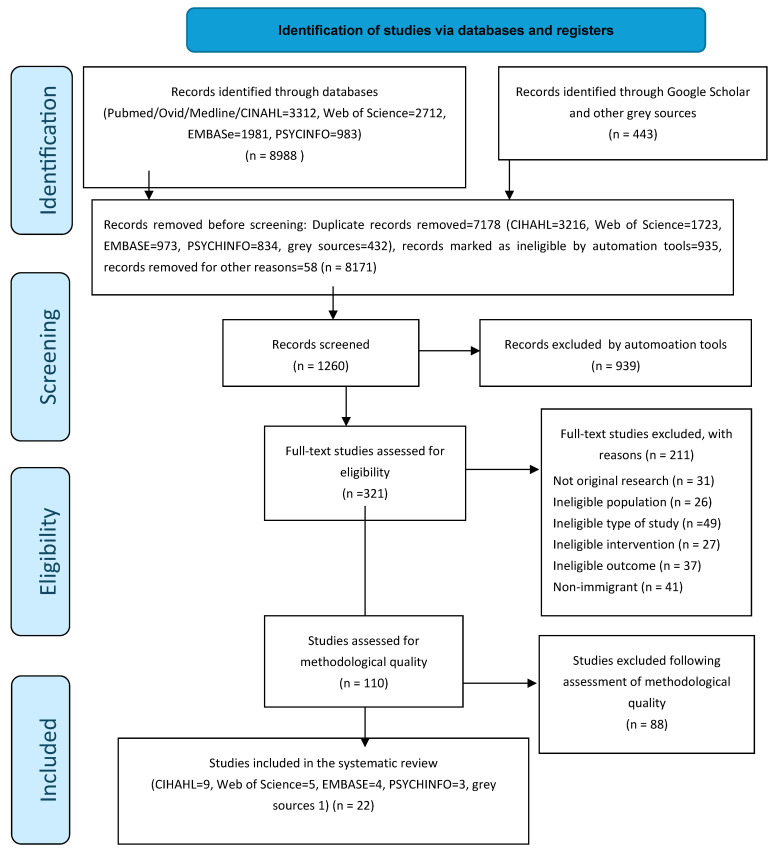
PRISMA 2020 flow diagram for new systematic reviews, which included searches of databases and registers only [13].

A PRISMA diagram was created, showing the details of the studies included and excluded at each stage of the study selection process (Figure 1).

### 2.7. Data Extraction

Covidence software (version 2, 2020, Veritas Health Innovation, Melbourne, Australia. Melbourne, Australia, https://www.covidence.org/, accessed on 21 December 2020) was used to deduplicate reviews and to extract data on study setting and design and intervention characteristics. We used Covidence software for conducting systematic reviews to streamline workflows for the research teams. Covidence was used to collaborate with multiple researchers to work on the same systematic review, enhancing team productivity. Covidence supported various stages of the systematic review process, including screening, data extraction, and quality assessment, making it a complete tool for researchers. Data extraction was transformed and coded to facilitate each element of the discussion, integrate the existing evidence, and answer the review questions [15]. Using Covidence software, a review matrix was generated to maximize efficiency and extract the pertinent study findings, specifically from each citation’s results and discussion sections (Table S2). All study findings from the included citations were coded for analysis as textual descriptions. Qualitative data comprised themes and subthemes with corresponding quotations and narrative interpretations to answer the review questions. Three independent reviewers (M.S.D., J.D.B, and A.A.N.) reviewed qualitative studies, and a fourth reviewer (A.S.) used the standardized JBI data extraction tool (Supplementary Fille S1 to S4) in JBI SUMARI [15]. Two of the reviewers (M.S.D. and A.A.N.) assessed the published paper’s trustworthiness, relevance, and results with the qualitative data extraction tool.

### 2.8. Data Transformation

In content analysis, themes or categories were developed a priori (i.e., before integration), and then all extracted data were coded according to these categories or themes [15]. This coding was followed by creating tabulations of frequency counts to identify key findings [15]. This count involved transformation into textual descriptions or narrative interpretation of the qualitative results in a way that answered the review questions by repeated detailed examination.

### 2.9. Data Synthesis and Integration

This review followed a qualitative approach to synthesis and integration according to the JBI methodology using JBI SUMARI. This approach involved separate qualitative synthesis and integration of the resultant qualitative evidence [15]. Qualitative research findings were pooled using JBI SUMARI with the meta-aggregation approach [15]. This approach involved the aggregation or synthesis of findings to generate a set of statements representing that aggregation by assembling the findings and categorizing these findings based on similarity in meaning. These categories are then subjected to a synthesis to produce a comprehensive set of synthesized findings that were used as a basis for evidence-based practice.

### 2.10. Registration

Registration: Open Science Framework DOI 10.17605/OSF.IO/TXQR5. https://osf.io/x26cn (accessed on 14 February 2025).

## 3. Results

### 3.1. Study Inclusion

**Figure 2:**
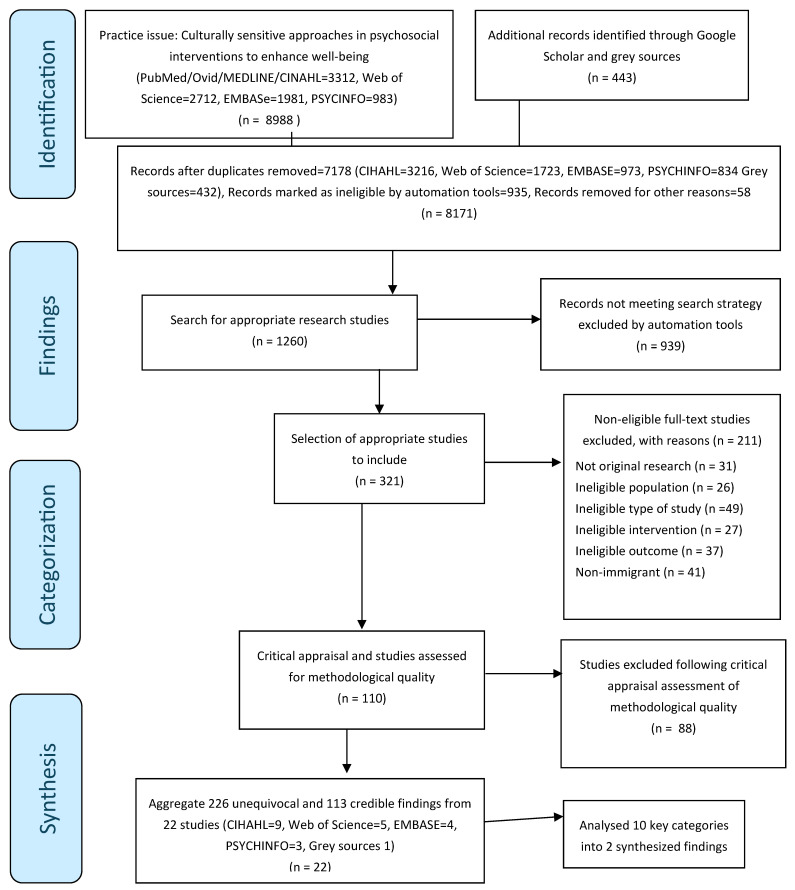
Meta-aggregative overview flowchart [14].

A total of 9431 articles were identified through 8 database searches. After removing 8171 duplicate publications, 321 titles and abstracts were screened for eligibility, and 211 articles were excluded for not meeting the criteria. After 110 full-text reviews for methodological quality, 22 studies were included in the data analysis and synthesis (Figure 1 and Figure 2).

### 3.2. Participant

Study sample sizes ranged from 10 to 5146 participants aged 18 years and older (Table S3). Most cultural care and psychosocial well-being included minority, ethnic, underserved, and low-income immigrant women.

### 3.3. Content Analysis

Content analysis showed two synthesized findings and six categories based on 226 contextual statements and 113 codes (Table S4). Content analysis of experiences of cultural considerations in care revealed themes such as the development of culturally responsive care models; barriers and gaps in culturally responsive care in rural communities; and patient information, education and culturally responsive care. The findings of experiences of psychosocial well-being of immigrants revealed themes such as cultural stigma, self-perception of access, use, and the role of healthcare providers; the impact of cancer and linguistically appropriate care; and challenges with psychosocial well-being and culturally responsive care (Table S4).

### 3.4. Synthesized Finding 1: Experiences of Cultural Considerations in Care

#### 3.4.1. Theme 1: Development of Culturally Responsive Care Models

The literature highlighted the need for culturally responsive care to enhance cancer care and screening for immigrant women. Culturally responsive care is termed as intentionally recognizing and respecting a person’s cultural background, beliefs, values, and experiences and incorporating these into their care and treatment [17]. Cultural issues include the lack of awareness and fear regarding cancer screening, language, barriers, and the unfamiliarity with and lack of trust in the healthcare system among Asian American women who are less acculturated and newly immigrated [18]. The women’s health educator’s accompaniment to screening was a valued aspect during the screening experience as she provided emotional and language support to immigrant women, enabling them to have the procedure explained thoroughly and ask questions via interpretation and, thereby, understand the process of screening, findings, or need for follow-up [19]. In conjunction with understanding gender-based needs, diversity and heterogeneity within ethnic groups are also crucial for tailoring care to the specific needs of breast cancer survivors [20]. These cultural differences encompass physical and emotional well-being, social network size and composition, cultural health beliefs, and doctor–patient communication [20]. Numerous barriers hinder access to culturally responsive and linguistically appropriate psychosocial interventions, including limited availability, accessibility, and acceptability [20].

#### 3.4.2. Theme 2: Barriers and Gaps in Culturally Responsive Care in Rural Communities

The research showed that participation in medical treatments [21] facilitated breast cancer survivorship. Experience with digital technology for health [22] was a facilitator of breast cancer treatment. Perspectives on survivorship care plan content and delivery [23,24,25] were facilitators of breast cancer care. Screening services for women [19] were facilitators of breast cancer care. Evolving needs throughout their cancer journey, tailoring information to patients’ needs, and transitioning to follow-up care were important facilitators of breast cancer post-treatment [25]. Participants’ preconceptions and assumptions of breast cancer support groups [26], engagement and empowerment, use of social support and other coping behaviours [27], and educational support through professionally led support groups [26,28] were facilitators of breast cancer care. The usefulness of intervention components [29] and writing as a process [30] were important facilitators of breast cancer treatment. The types of psychosocial distress, anxiety, and uncertainty that minority breast cancer survivors experience and the lack of adequate support and guidance emphasized the value of community-based, population-specific cancer support programs, such as the Sisters Network [24]. Healthcare professionals must tailor care to meet individual’s unique needs, beliefs, and practices from different cultural backgrounds [25,31]. Key aspects include understanding cultural beliefs, coping strategies, family support, spirituality, and addressing barriers to healthcare access [31]. Recognizing and adapting to the evolving needs at different stages of the cancer journey, while considering the influence of personal and social contexts, are a key facilitator in providing care [25].

#### 3.4.3. Theme 3: Patient Information, Education, and Culturally Responsive Care

The literature shed light on the need for increased diversity within the healthcare system and staffing to enhance language skills, educational programs, and cultural competence to support immigrant women [31,32]. The fostering of a caring and trusting environment in which immigrant women can share their experiences, feelings, and concerns regarding their care is beneficial in empowering the women and further improving their psychosocial outcomes [19]. The position of the cancer care program’s role in informing and empowering patients positively impacts the quality of life of cancer patients by creating a culturally responsive care plan [33]. The benefits of peer support and the integration of technology to provide this support [33] have positive benefits in helping patients improve their motivation, proactivity, and resilience in pursuing recovery and psychosocial well-being [22]. Chinese Australian women use strategies to adjust to their breast cancer experience, such as dietary changes, exercise, spirituality, and cognitive reframing [27]. Developing culturally responsive self-management resources for this minority group has potential empowering and beneficial results for their journey [27]. Using a mobile phone app and telephone coaching to deliver empowering and motivating care has proven beneficial [28]. Cancer treatment significantly affects the self-efficacy, emotional well-being, and body image of Spanish-speaking breast cancer survivors, and a technology-driven intervention program helps reduce stress and enhance positivity about physical appearance and weight for the participants [28]. Motivation and empowerment are common themes discussed as program outcomes that enable confident, individualized self-care [28]. Despite the stigma, challenges, and hardships faced by cancer survivors, the participants’ ability to try to find appropriate care and positive meaning in their experiences emphasizes their resilience [7]. While individuals may employ diverse methods influenced by their cultural beliefs and values, the common theme of resilience reflects the concept of post-traumatic growth, referring to the positive psychosocial change experienced because of the struggle with challenging circumstances [7].

### 3.5. Synthesized Finding 2: Experiences and Psychosocial Well-Being of Immigrants

#### 3.5.1. Theme 4: Cultural Stigma and Self-Perception of Access, Use, and the Role of Healthcare Providers

Cultural identity and power are outlined as acknowledging and respecting the patient’s cultural identity and background and recognizing the power dynamics and potential biases that may exist within caring relationships. Latina breast cancer survivors agreed that tailored verbal and printed education was vital during their transition process, keeping the women engaged and empowering participants to share what they learned [28]. Chinese Australian women reported difficulties with managing the side effects of treatment and noted that these side effects were disruptive to their daily lives and functioning [27]. Latina immigrant breast cancer survivors commented that the cultural change impacted their quality of life, as they often felt rushed and perceived stigma, shame, secrecy, embarrassment, and a sense of urgency in terms of making medical decisions [34]. Latina immigrant breast cancer survivors, even those who spoke English, faced language challenges that complicated their survivorship care, and many experienced problems with the interpretation process [34,35]. South Asian breast cancer survivors felt that specific physical activities should be communicated so that they can overcome fatigue, depression, and weight gain during active treatment and afterwards [36]. Black and South Asian women born overseas reported needing a relative with them at appointments to enable them to understand the context of conversations with healthcare professionals [37]. Indian and Pakistani women who were born overseas and required accompaniment to appointments often tended to fall into the lower socioeconomic position category [37]. White British women were more inclined to engage in interactive dialogue and communication with their healthcare professionals during follow-up appointments, in contrast to Black African women who were more likely to rely on their healthcare professionals to lead the dialogue [37]. Participants expressed a sense of identity, such as their roles as mothers, wives, and caregivers, shaped by their ethnicity, acculturation, socioeconomic status, education, cultural factors, and cultural competence [31]. The impact of breast cancer on a woman’s sense of identity is multifaceted as they navigate their roles as mothers, wives, and caregivers; their identity becomes intertwined with their cancer experience [31]. Cultural factors, such as ethnicity, acculturation, socioeconomic status, and education, further shape their self-perception [31]. Coping with these identity changes and challenges due to breast cancer is an essential aspect of their psychosocial well-being and influences the care they receive [31]. The sense of identity of cancer patients is dynamic and influenced by various factors, such as cultural perceptions of cancer, treatment, and support groups [26]. The culturally dynamic perceptions of support groups may challenge the sense of identity and agency, and some patients may not see themselves as belonging to a community of cancer survivors [26].

#### 3.5.2. Theme 5: Impact of Cancer and Linguistically Appropriate Care

A lack of fluency and discomfort in speaking English among Asian American women contributes to a lack of active involvement in their care [18]. Latinas believed that medical staff made assumptions based on their inability to speak English and manner of dress, and Asian American survivors felt that medical staff gave more information to those who were more educated about breast cancer [31]. There is a need for breast cancer survivors’ minority-specific considerations when providing information to patients, as patients often have information gaps, confusion, and dissatisfaction with communication from their primary care providers [24]. Discrepancies in the understanding between patients and healthcare providers are a key driver of the information gaps that patients experience [23,31]. Factors such as definitions of the care received and the terminology used to capture the ongoing nature of their treatment are examples of discrepancies that display a need for linguistically appropriate care [23].

#### 3.5.3. Theme 6: Challenges with Psychosocial Well-Being and Culturally Responsive Care

The literature highlighted various challenges in the implementation of culturally responsive care. For example, breast cancer survivors who were personally less satisfied with the survivorship care plan were less likely to share the information with the healthcare team, users did not believe that it was relevant to their current care (20%), and users were concerned that the healthcare team would not care about their care plan [33]. Asian Americans reported limited English proficiency as a barrier to accessing health information and quality of healthcare and doctor–patient communication and medical decisionmaking, such as insurance and medical costs, limited time with providers, and lack of medical translation services [20]. Socioeconomic and cultural factors and systemic problems in the healthcare system were the major barriers to access and quality of care among Asian American women [31]. The level of acculturation affects the seeking of medical care. Latinas born in the United States were more willing to adopt Western culture, perform self-breast exams, obtain mammograms, adhere to modern medical treatment, and be watchful of their health [31]. The Latino community hesitates to access medical resources due to language barriers and logistical challenges associated with negotiating the complex healthcare system, what information or services to utilize, and how to find them [31]. Also, 92% of users reported that the care plan increased their knowledge of the possible long-term and late effects of their cancer, and 84% reported that the care plan improved their knowledge of what medical tests should be conducted as part of the follow-up care for their cancer [33]. South American breast cancer survivors felt they should receive a list of resources, including those for reconstruction, reproduction, sexuality, nutrition, exercise, and prevention, which should be explained at discharge [36]. Women overwhelmingly said they wanted someone to talk to post-treatment and expected that their breast cancer nurse would fulfill their role [37]. The challenges described by participants were a lack of health insurance and an unfamiliarity with the healthcare system, and a few needed to be made aware that they were eligible for health insurance [7]. Other participants referred to difficulties in obtaining information about the medical and insurance system, such as who to approach or contact to learn more about the reimbursement process for medical expenses [7]. Policies must address healthcare disparities and provide adequate support for cancer survivors from diverse backgrounds [31]. Policies could involve improving access to healthcare resources, enhancing patient education about the disease, or developing support programs tailored to the needs of different ethnic groups [31]. Latina breast cancer survivors’ experiences of stigma, shame, and embarrassment as immigrants affect their survivorship experiences [34]. Factors such as language barriers, social isolation, and changes in social habits reflecting acculturation may pose additional challenges for these women [34,38]. Experiences of culturally responsive care, access to insurance and benefits, levels of knowledge and practices, availability of language-appropriate care, access to healthcare services, and the overall quality of care influenced the quality of life for immigrant breast cancer survivors [39,40].

## 4. Discussion

Based on our review of the literature, we note that experts in the field raise similar concerns about culturally sensitive approaches in psychosocial interventions to enhance the well-being of immigrant adults diagnosed with breast cancer. The discussion helped to identify strengths, best practices, insights into health equity, and policy implications and contextualize challenges across geographies and diverse populations so that we can offer valuable perspectives for healthcare providers in Canada. Although there is evidence for psychosocial well-being in some specific domains or aspects of care, the relationships between the psychosocial well-being of immigrant adults diagnosed with breast cancer and recommendations remain fragile. This review of the literature emphasizes that the need for the psychosocial well-being of immigrant adults is an ongoing issue for this population despite being highlighted. We also found that there are no interventions focused on addressing the psychosocial well-being of immigrant adults diagnosed with breast cancer.

Healthcare research to improve the cultural care of immigrant adults may be more challenging than improving access to supportive programs for immigrant women with breast cancer. Immigrant women living with breast cancer perceive communication and participation, doctor–patient relationships, knowledge of services, trust, and respect as crucial elements in their experience. These women often feel vulnerable, experiencing a sense of isolation, being stigmatized, ashamed, and embarrassed, lacking information, and facing challenges related to family commitments and cultural barriers [41]. Changes in sociocultural behaviours and the challenges of understanding and speaking English, combined with a breast cancer diagnosis and treatment, can exacerbate feelings of worry, fear, and anxiety. These issues, along with feelings of stigmatization, may deter immigrant women from seeking adequate support and affect their ability to cope with stressors and overall well-being [42]. Social justice and health equity policies for breast cancer survivors are crucial for seeking cancer-supportive care [41,42]. This health-seeking behaviour prompts co-creating and equity-oriented care requirements through culturally and linguistically responsive services. Healthcare providers must be aware of cultural values and practise humility to enhance cultural care and the quality of care for immigrant women [43]. These studies do not refer to culturally and linguistically appropriate health and wellness services. There is an evident paucity of research on this topic, which prompts the need to provide community-supportive care to immigrant women who are uncertain about talking with their healthcare providers due to a language hurdle.

In our review, we found that in Southeast Asian and Arab cultures, emotions are suppressed to maintain familial and societal harmony, inhibiting the individual’s capability to assert one’s beliefs through emotional expression. Breast cancer care issues could be different for immigrant women due to different perceptions of cancer-related stigma and social, cultural, and spiritual beliefs and values. Education, access, and culture-specific barriers like breast exposure, uneasiness with one’s own body, and social, environmental, and religious beliefs influenced breast cancer screening and prevention [44]. Language is a cost-effective approach to navigating cancer care. Cultural and linguistically appropriate care is a set of policies enabling people to communicate efficiently with healthcare providers and for ensuring responsible communication with immigrants. Culture and language are important components in accessing and using healthcare and affect the person’s ability to navigate, support, communicate, and make decisions [43,44]. The review findings demonstrated that some women were less informed and prepared for the physical and psychological cancer treatment-related side effects that emerged during the cancer treatment process, post-treatment, and the long-term aftermath.

We identified that limited English proficiency and a lack of understanding about navigating healthcare services were challenging, and these caused emotional distress and potential health consequences for women with breast cancer. This systematic review has identified many categories of experiences of the cultural care of immigrant adults living with breast cancer. The primary outcome targeted was the quality of life during cancer treatment and post-treatment. In general, limited evidence suggests that cultural care and psychosocial well-being improve patient-oriented outcomes in women with breast cancer. The main barriers were accessing health information, a lack of patient–physician communication and decision-making support, and a lack of medical translation services, which were associated with a lack of social support and adopting recommendations.

Our work echoes previous reviews by conducting searches across multiple electronic databases, thereby extending the search period to 20 years. Additionally, this review included an evaluation of the quality of the research conducted. This review identified an increase in the number of cultural care and support groups for breast cancer, including the number of studies that reviewed cultural care for improving psychosocial well-being in under-represented and underserved populations. The research that has evaluated the effectiveness of cultural care and psychosocial well-being is of moderate quality, and the findings are mixed.

Limitations: We have limited the scope of the literature and excluded our ability to analyze the effect of the COVID-19 pandemic and the long-term effects of psychosocial well-being in the reported outcomes and draw tangible or directional conclusions. The findings of this review are limited by heterogeneity in terms of delivery, goals, services, healthcare providers, intended audiences, and targeted outcomes. This study limits the breadth of information synthesized from selected studies published in multiple languages. Some non-peer-reviewed studies may be missed in the evolving literature on psychosocial well-being.

## 5. Conclusions

Our findings highlight that psychosocial well-being has the potential to enhance cultural care for immigrant women living with breast cancer. These interventions may normalize the use of such services, provide information about their benefits and where to access them, and confirm the availability of culturally congruent counsellors and interpreters. Healthcare providers can benefit from training that emphasizes culturally responsive communication skills. Such training can teach them how to elicit psychosocial needs and knowledge gaps and address patients’ concerns effectively. Furthermore, psychosocial service providers can benefit from culturally oriented training to deliver culturally responsive services. The training should address concerns related to language interpretation within the context of psychosocial oncology services. Healthcare institutions can contribute to improving cultural competency by implementing policies, processes, and programs. This includes developing and supporting culturally responsive training for providers and support staff, maintaining a diverse workforce of bilingual language and psychosocial providers and support staff, and offering language assistance to patients and caregivers with limited English proficiency. Further strategies involve translating all materials to the patient’s preferred languages, conducting ongoing assessments of policies regarding culturally and linguistically responsive care, and fostering partnerships with the community to ensure culturally and linguistically appropriate psychosocial services. Collaboration with community clinics and programs can facilitate effective transitions of psychosocial care. Implementing a team approach, care management, continuity of care, and health navigation can address logistical, psychosocial, socioeconomic, and cultural needs. Additionally, maintaining cultural and community advisory boards can aid in planning and monitoring programs and policies, contributing to continuous improvement in cultural competency in healthcare approaches.

This study revealed several challenges that intersect to shape the access, utilization, and effectiveness of healthcare services across the cancer continuum. These challenges include pre-existing individual characteristics, interactions with healthcare facilities facilitated by physicians and healthcare providers, sociocultural expectations, and gender norms. For immigrant breast cancer survivors, experiences of culturally responsive care, insurance and benefits, lack of knowledge and practices, language-appropriate care, access to care, and quality of care collectively influence their quality of life. Factors such as a lack of knowledge about healthcare providers, contact with cancer care services, language barriers, English language proficiency, cultural factors, lack of support, unmet needs, absence of survivorship and long-term care, and the cost of extended healthcare services influence the meaningfulness of cultural care in improving the quality of life for immigrant adults living with breast cancer. In addition, a lack of understanding of the English language and navigating healthcare services leads to increased psychosocial and emotional distress for immigrant women. Establishing culturally and linguistically appropriate care is crucial for developing a trusting relationship with healthcare providers, especially for decision making regarding treatment. While there is growing support for cultural care and psychosocial well-being in breast cancer, additional research is needed to determine the effectiveness of cultural care and psychosocial well-being in breast cancer treatment and post-treatment. Future research should compare different models of breast cancer care using patient-oriented outcomes and adhere to recommended metrics, cultural care, and psychosocial outcomes during cancer treatment and post-treatment. A more rigorous evaluation of cultural care and psychosocial well-being is needed. Studies should recruit larger, ethnically diverse samples and evaluate whether cultural care and psychosocial well-being equally benefit all ethnic groups and increase the generalizability of the findings. Future studies should include cohort-based prospective follow-ups of people with breast cancer post-treatment and track the recurrence of cancer and survival data. Future research should examine the processes by which cultural care and psychosocial well-being improve women’s goals to determine the components of breast cancer care that are most likely to be associated with timely, high-quality, and recommended treatment, post-treatment care, and survival from breast cancer.

## 6. Implications

This study’s findings have several implications for both practice and research; as such, we offer the following recommendations.

### 6.1. Practice

From the practice perspective, we emphasize the importance of addressing the cultural-specific needs of breast cancer survivors, emphasizing communication and ongoing support throughout the entire continuum from screening and diagnosis to treatment and survivorship. This study proposes that culturally specific policies need to address healthcare discrepancies and provide adequate support for cancer survivors, such as improving access to healthcare resources, enhancing education and health promotion, or developing support programs tailored to the needs of different ethnic and cultural groups. This review paper suggests future directions for improving collaborations between public health, primary care, and community services. It also recommends involving culturally diverse immigrant women in the design and evaluation of cancer care services and resources. Introducing culturally responsive screening invitations tailored to encourage participation becomes imperative. Family support is important for providing transport for appointments and language interpretation. Spirituality support is central to enhancing spiritual beliefs and practices as a source of comfort, confidence, and coping mechanisms. This practice helps women with traditional values and support groups to recognize spiritual resources. There is a need to ensure culturally responsive, safe, equitable, affordable, and accessible care while considering the type of health insurance and the state of their finances, which is linked to a better quality of care. Enhancing timely access and connection to survivorship for cancer patients and their families leads to a better quality of care. Patient–physician relationships, communication, and shared decision making are influential factors in adopting health promotion practices to improve the quality of care. Raising awareness, knowledge, and information about breast cancer treatment and follow-up affects the quality of care. There is a need for the coordination and integration of cancer-supportive care to enhance patient-oriented outcomes, psychosocial safety, information about healthcare services, and psychosocial support. Leveraging sociocultural determinants to better integrate cancer care can enhance psychosocial well-being. Therefore, cultural competence can be enhanced by increasing the uptake of formal cultural competency training among healthcare providers.

### 6.2. Research

For research, future practice-oriented studies should explore physical, emotional, and psychological factors that can significantly impact the quality of life during breast cancer and its treatment. For immigrant women who are breast cancer survivors, enhancing their quality of life involves integrating research and building the capacity for participation. Developing informational, educational, and awareness materials about breast cancer that are culturally responsive and linguistically tailored can be particularly helpful for this specific cultural population. Breast cancer survivors and their families may require dedicated time to engage with healthcare professionals, discussing signs of recurrence, testing for metastasis, and available treatment options. Encouraging increased family and spouse participation can be achieved through initiatives such as improving health literacy, providing counselling, offering educational programs, facilitating referrals to healthcare services, implementing cultural care, and increasing navigation in the healthcare system. Establishing a reported outcome data platform that supports care decisions and serves as an engine for policy can actively engage individuals in their cancer care continuum. There is a notable emphasis on self-management and building the capacities of immigrant women to be better prepared for breast cancer treatment, which includes managing symptoms, handling adverse reactions, and individualizing care plans. Therefore, expanding digital health literacy solutions becomes crucial to improving access and relevance for individuals, effectively addressing the unique needs of immigrant women with breast cancer.

## Data Availability

Open Science Framework DOI 10.17605/OSF.IO/TXQR5. https://osf.io/txqr5/ accessed on 21 December 2021.

## Supplementary Materials

The following supporting information can be downloaded at https://www.mdpi.com/article/10.3390/ijerph22030335/s1, Supplementary File S1. Database(s): Ovid MEDLINE(R) and In-Process & Other Non-Indexed Citations 2001 to 21 December 2021, and search strategy: BCS PubMed/Ovid/MEDLINE TO CINAHL 2021-12-22. Supplementary File S2. Database(s): Ovid MEDLINE(R) and In-Process & Other Non-Indexed Citations 2001 to 21 December 2021, and search strategy: BCS PubMed/Ovid/MEDLINE TRANSLATION TO EMBASE 2021-12-22. Supplementary File S3. Database(s): Ovid MEDLINE(R) and In-Process & Other Non-Indexed Citations 2001 to 21 December 2021, and search strategy: BCS PubMed/Ovid/MEDLINE to PSYCINFO 2021-12-22. Supplementary File S4. Database(s): Ovid MEDLINE(R) and In-Process & Other Non-Indexed Citations 2001 to 21 December 2021, and search strategy: BCS PubMed/Ovid/MEDLINE TRANSLATION TO WEB OF SCIENCE 2021-12-22. Supplementary File S5: PRISMA 2020 Checklist. Table S1. Critical appraisal results of eligible studies; Table S2. JBI Critical Appraisal Checklist for Qualitative Systematic Reviews; Table S3. Data extraction instrument; Table S4. Meta-aggregation of synthesized findings, context, codes and categories. Reference [45] is cited in the supplementary materials.
