# Supplementary material for: Culturally Sensitive Approaches in Psychosocial Interventions to Enhance Well-Being of Immigrant Adults Diagnosed with Breast Cancer: A Systematic Review
Source: Int J Environ Res Public Health. 2025 Feb 25;22(3):335. doi: 10.3390/ijerph22030335 (PMC11942024; doi:10.3390/ijerph22030335)
Supplement: Supplementary file 1 [file ijerph-22-00335-s001.zip › ijerph-3436135-supplymentary File S1-S4.pdf]

Supplementary File S1. Database(s): Ovid MEDLINE(R) and In-Process & Other Non-Indexed Citations 2001 to December 21, 2021 and Search Strategy: BCS PubMed/Ovid/MEDLINE TO CINAHL 2021-12-22

#	MEDLINE	CINAHL	Search
1	Female/	(MH "Female")	
2	(female* or wom?n or mother* or grandmother* or girl*).mp.	(female* or women or woman or mother* or grandmother* or girl*)	
3	1 or 2	S1 or S2	
4	Breast Neoplasms/	(MH "Breast Neoplasms") OR (MH "Carcinoma, Ductal, Breast") OR (MH "Hereditary Breast and Ovarian Cancer Syndrome")	S4
5	"Hereditary Breast and Ovarian Cancer Syndrome"/		
6	Breast Cancer Lymphedema/		
7	Breast Carcinoma in Situ/		
8	Breast Neoplasms/		
9	Carcinoma, Ductal, Breast/		
10	Carcinoma, Lobular/		
11	Inflammatory Breast Neoplasms/		
12	exp Mastectomy/	(MH "Mastectomy") OR (MH "Lumpectomy") OR (MH "Prophylactic Mastectomy") OR (MH "Metastasectomy") OR (MH "Lymph Node Excision") OR (MH "Axillary Lymph Node Dissection")	S5
13	Triple Negative Breast Neoplasms/		
14	Unilateral Breast Neoplasms/		
15	or/4-14		
16	((brca or mastectomy* or (breast* or mammary)) adj4 (adenocarcinoma* or cancer* or carcinoma* or metasta* or malignan* or neoplasm* or tumor* or tumour*)).mp.	((brca or mastectomy* or (breast* or mammary)) N4 (adenocarcinoma* or cancer* or carcinoma* or metasta* or malignan* or neoplasm* or tumor* or tumour*))	S6
17	15 or 16	S4 or S5 or S6	
18	Breast Diseases/	(MH "Breast Diseases")	
19	Breast/	(MH "Breast") OR (MH "Breast Tissue Density") OR (MH "Nipples")	
20	Mammary Glands, Human/		
21	Nipples/		
22	or/18-21		

23	(breast* or mammary).mp.	(breast* or mammary)
24	22 or 23	
25	exp Neoplasms/	(MH "Neoplasms+")
26	24 and 25	
27	17 or 26	
28	3 and 27	
29	Peer Group/	(MH "Peer Group") OR (MH "Support Groups")
30	Self-Help Groups/	
31	29 or 30	
32	((emotional or community or grassroots or psychosocial or psychologic* or peer* or self-help or social) adj3 (group* or support* or care or caring)).mp.	((emotional or community or grassroots or psychosocial or psychologic* or peer* or self-help or social) N3 (group* or support* or care or caring))
33	31 or 32 (cultur* or psychosocia*)	
34	28 and 33 (well-bein* or immigran*)	
35	attitude to death/ or uncertainty/	(MH "Attitude to Illness") OR (MH "Attitude to Death") OR (MH "Attitude to Health") OR (MH "Attitude to Life") OR (MH "Uncertainty")
36	exp adaptation, psychological/	
37	exp attitude to health/	
38	exp *Consumer Satisfaction/	
39	Depression/	
40	Exp Emotions/	
41	life change events/	
42	Patient Preference/ Adult	
43	exp Patients/px [Psychology]	

44	personal autonomy/	
45	*"Quality of Life"/ Intervention	
46	self care/	
47	self concept/	
48	exp self-efficacy/	
49	stress, psychological/	
50	coping.ti,ab.	
51	empowerment.tw.	
52	"informed choice".ti,ab.	
53	(patient adj3 (attitude$ or preference$)).ti,ab.	
54	"patient satisfaction".ti.	
55	((client* or consumer$ or famil$ or parent$ or patient$ or spouse$) adj (activation or attitude$ or desir$ or involvement or perspective$ or preference$ or view$)).ti,ab.	
56	(QoL or "quality of life").ti.	
57	self-management.ti.	
58	("self-perception" or "self-concept").ti,ab.	
59	or/35-58	
60	34 and 59	
61	Total records	3312

Supplementary File S2. Database(s): Ovid MEDLINE(R) and In-Process & Other Non-Indexed Citations 2001 to December 21, 2021
Search Strategy: BCS PubMed/Ovid/MEDLINE TRANSLATION TO EMBASE 2021-12-22

#	MEDLINE	EMBASE
1	Female/	divorced woman/ or<br>female by marital status/ or<br>female/ or<br>married woman/ or<br>single woman/ or<br>widow/
2	(female* or wom?n or mother* or grandmother* or girl*).mp.	(female* or wom?n or mother* or grandmother* or girl*).mp.
3	1 or 2	
4	Breast Neoplasms/	basal like breast cancer/ or<br>breast adenocarcinoma/ or<br>breast cancer molecular subtype/ or<br>breast cancer/ or<br>breast carcinogenesis/ or<br>breast carcinoma/ or<br>breast sarcoma/ or<br>carcinoma/ or<br>inflammatory breast cancer/ or<br>lobular carcinoma/ or<br>metastatic breast cancer/ or<br>paget nipple disease/<br>phyllodes tumor/
5	"Hereditary Breast and Ovarian Cancer Syndrome"/	
6	Breast Cancer Lymphedema/	breast cancer-related lymphedema/
7	~~Breast Carcinoma in Situ/~~	
8	~~Breast Neoplasms/~~	
9	Carcinoma, Ductal, Breast/	
10	Carcinoma, Lobular/	lobular carcinoma/ or
11	Inflammatory Breast Neoplasms/	
12	exp Mastectomy/	
13	Triple Negative Breast Neoplasms/	

14	Unilateral Breast Neoplasms/	
15	or/4-14	
16	((brca or mastectomy* or (breast* or mammary)) adj4 (adenocarcinoma* or cancer* or carcinoma* or metasta* or malignan* or neoplasm* or tumor* or tumour*)).mp.	
17	15 or 16	
18	Breast Diseases/	breast tumor/ or
19	Breast/	
20	Mammary Glands, Human/	
21	Nipples/	
22	or/18-21	
23	(breast* or mammary or nipple*).mp.	(breast* or mammary or nipple*).mp.
24	22 or 23	
25	exp Neoplasms/	
26	24 and 25	
27	17 or 26	
28	3 and 27	
29	Peer Group/	
30	Self-Help Groups/	
31	29 or 30	
32	((emotional or community or grassroots or psychosocial or psychologic* or peer* or self-help or social) adj3 (group* or support* or care or caring)).mp.	
33	31 or 32 (cultur* psychosocia*)	
34	28 and 33 (immigran*well-bein*)	
35	attitude to death/	
36	exp adaptation, psychological/	
37	exp attitude to health/	
38	exp *Consumer Satisfaction/	
39	Depression/	122158
40	exp Emotions/	68387
41	life change events/	22824
42	Patient Preference/ Adult	8911
43	exp Patients/px [Psychology]	17464
44	personal autonomy/	17225

45	*"Quality of Life"/ Intervention	91412
46	self care/	33484
47	self concept/	57268
48	exp self-efficacy/	20832
49	stress, psychological/	122329
50	coping.ti,ab.	54165
51	empowerment.tw.	12003
52	"informed choice".ti,ab.	1525
53	(patient adj3 (attitude$ or preference$)).ti,ab.	13298
54	"patient satisfaction".ti.	6213
55	((client* or consumer$ or famil$ or parent$ or patient$ or spouse$) adj (activation or attitude$ or desir$ or involvement or perspective$ or preference$ or view$)).ti,ab.	45577
56	(QoL or "quality of life").ti.	70627
57	self-management.ti.	6736
58	("self-perception" or "self-concept").ti,ab.	8760
59	or/35-58	1046732
60	34 and 59	2484
61	Total records	1981

Supplementary File S3. Database(s): Ovid MEDLINE(R) and In-Process & Other Non-Indexed Citations 2001 to December 21, 2021
Search Strategy: BCS PubMed/Ovid/MEDLINE to PSYCINFO 2021-12-22

#	Searches	Psycinfo
1	Female/	female attitudes/ or human females/ or daughters/ or mothers/ or wives/ or working women/
2	(female* or wom?n or mother* or grandmother* or girl*).mp.	
3	1 or 2	
4	Breast Neoplasms/	
5	"Hereditary Breast and Ovarian Cancer Syndrome"/	
6	Breast Cancer Lymphedema/	
7	Breast Carcinoma in Situ/	
8	Breast Neoplasms/	
9	Carcinoma, Ductal, Breast/	
10	Carcinoma, Lobular/	
11	Inflammatory Breast Neoplasms/	
12	exp Mastectomy/	
13	Triple Negative Breast Neoplasms/	
14	Unilateral Breast Neoplasms/	
15	or/4-14	
16	((brca or mastectomy* or (breast* or mammary)) adj4 (adenocarcinoma* or cancer* or carcinoma* or metasta* or malignan* or neoplasm* or tumor* or tumour*)).mp.	
17	15 or 16	
18	Breast Diseases/	
19	Breast/	
20	Mammary Glands, Human/	
21	Nipples/	
22	or/18-21	
23	(breast* or mammary).mp.	
24	22 or 23	
25	exp Neoplasms/	
26	24 and 25	

27	17 or 26	
28	3 and 27	
29	Peer Group/	
30	Self-Help Groups/	
31	29 or 30	
32	((emotional or community or grassroots or psychosocial or psychologic* or peer* or self-help or social) adj3 (group* or support* or care or caring)).mp.	
33	31 or 32 (cultur* or psychosocia*)	
34	28 and 33 (well-bein* or immigran*)	
35	attitude to death/ or<br>uncertainty/	
36	exp adaptation, psychological/	
37	exp attitude to health/	
38	exp *Consumer Satisfaction/	
39	Depression/	
40	exp Emotions/	
41	life change events/	
42	Patient Preference/ Adult	
43	exp Patients/px [Psychology]	
44	personal autonomy/	
45	*"Quality of Life"/ Intervention	
46	self care/	
47	self concept/	
48	exp self-efficacy/	
49	stress, psychological/	
50	coping.ti,ab.	
51	empowerment.tw.	
52	"informed choice".ti,ab.	
53	(patient adj3 (attitude$ or preference$)).ti,ab.	
54	"patient satisfaction".ti.	
55	((client* or consumer$ or famil$ or parent$ or patient$ or spouse$) adj (activation or attitude$ or desir$ or involvement or perspective$ or preference$ or view$)).ti,ab.	
56	(QoL or "quality of life").ti.	
57	self-management.ti.	

58	("self-perception" or "self-concept").ti,ab.	
59	or/35-58	
60	34 and 59	
61	Total records	983

Supplementary File S4. Database(s): Ovid MEDLINE(R) and In-Process & Other Non-Indexed Citations 2001 to December 21, 2021
Search Strategy: BCS PubMed/Ovid/MEDLINE TRANSLATION TO WEB OF SCIENCE 2021-12-22

#	MEDLINE	Web of Science
1	Female/	
2	(female* or wom?n or mother* or grandmother* or girl*).mp.	(female* or wom?n or mother* or grandmother* or girl*)
3	1 or 2	
4	Breast Neoplasms/	
5	"Hereditary Breast and Ovarian Cancer Syndrome"/	
6	Breast Cancer Lymphedema/	
7	Breast Carcinoma in Situ/	
8	Breast Neoplasms/	
9	Carcinoma, Ductal, Breast/	
10	Carcinoma, Lobular/	
11	Inflammatory Breast Neoplasms/	
12	exp Mastectomy/	
13	Triple Negative Breast Neoplasms/	
14	Unilateral Breast Neoplasms/	
15	or/4-14	
16	((brca or mastectomy* or (breast* or mammary)) adj4 (adenocarcinoma* or cancer* or carcinoma* or metasta* or malignan* or neoplasm* or tumor* or tumour*)).mp.	((brca or mastectomy* or (breast* or mammary)) SAME (adenocarcinoma* or cancer* or carcinoma* or metasta* or malignan* or neoplasm* or tumo$r*))
17	15 or 16	
18	Breast Diseases/	
19	Breast/	
20	Mammary Glands, Human/	
21	Nipples/	
22	or/18-21	
23	(breast* or mammary).mp.	
24	22 or 23	
25	exp Neoplasms/	
26	24 and 25	

27	17 or 26	
28	3 and 27	
29	Peer Group/	
30	Self-Help Groups/	
31	29 or 30	
32	((emotional or community or grassroots or psychosocial or psychologic* or peer* or self-help or social) adj3 (group* or support* or care or caring)).mp.	((emotional or community or grassroots or psychosocial or psychologic* or peer* or self-help or social) SAME (group* or support* or care or caring))
33	31 or 32 (cultur* or psychosocia*)	
34	28 and 33(well-bein* or immigran*)	
35	attitude to death/ or<br>uncertainty/	
36	exp adaptation, psychological/	
37	exp attitude to health/	
38	exp *Consumer Satisfaction/	
39	Depression/	
40	exp Emotions/	
41	life change events/	
42	Patient Preference/ Adult	
43	exp Patients/px [Psychology]	
44	personal autonomy/	
45	*"Quality of Life"/Intervention	
46	self care/	
47	self concept/	
48	exp self-efficacy/	
49	stress, psychological/	
50	coping.ti,ab.	coping.ti,ab.
51	empowerment.tw.	empowerment.tw.
52	"informed choice".ti,ab.	"informed choice".ti,ab.
53	(patient adj3 (attitude$ or preference$)).ti,ab.	patient adj3 (attitude$ or preference$)).ti,ab.
54	"patient satisfaction".ti.	"patient satisfaction".ti.

55	((client* or consumer$ or famil$ or parent$ or patient$ or spouse$) adj2 (activation or attitude$ or desir$ or involvement or perspective$ or preference$ or view$)).ti,ab.	((client* or consumer$ or famil$ or parent$ or patient$ or spouse$) SAME (activation or attitude$ or desir$ or involvement or perspective$ or preference$ or view$)) run in topic
56	(QoL or "quality of life").ti.	(QoL or "quality of life") run in ti
57	self-management.ti.	self-management run in ti.
58	("self-perception" or "self-concept").ti,ab.	("self-perception" or "self-concept") run in topic.
59	or/35-58	
60	34 and 59	
61	Total records	2712
