# Supplementary material for: Culturally Sensitive Approaches in Psychosocial Interventions to Enhance Well-Being of Immigrant Adults Diagnosed with Breast Cancer: A Systematic Review
Source: Int J Environ Res Public Health. 2025 Feb 25;22(3):335. doi: 10.3390/ijerph22030335 (PMC11942024; doi:10.3390/ijerph22030335)
Supplement: Supplementary file 1 [file ijerph-22-00335-s001.zip › ijerph-3436135-supplymentary Table S1-S4.pdf]

Table S1. Critical appraisal results of eligible studies.

Research Studies	Q1	Q2	Q3	Q4	Q5	Q6	Q7	Q8	Q9	Q10	Total Scores
Ashing-Giwa, K. T., Padilla, G., Tejero, J., Kraemer, J., Wright, K., Coscarelli, A., ... & Hills, D. (2004). Understanding the breast cancer experience of women: a qualitative study of African American, Asian American, Latina and Caucasian cancer survivors. *Psycho-Oncology: Journal of the Psychological, Social and Behavioral Dimensions of Cancer*, *13*(6), 408-428. (30)	Y	Y	Y	Y	Y	U	Y	Y	Y	Y	9
Burg, M. A., Lopez, E. D., Dailey, A., Keller, M. E., & Prendergast, B. (2009). The potential of survivorship care plans in primary care follow-up of minority breast cancer patients. *Journal of general internal medicine*, *24*(2), 467. DOI: 10.1007/s11606-009-1012-y. United States of America. (23)	Y	Y	U	Y	Y	Y	Y	Y	Y	Y	9
Levine, E. G., Aviv, C., Yoo, G., Ewing, C., & Au, A. (2009). The benefits of prayer on mood and well-being of breast cancer survivors. *Supportive Care in Cancer*, *17*, 295-306. (39)	Y	Y	Y	Y	Y	Y	Y	U	Y	Y	9
Lopez-Class, M., Perret-Gentil, M., Kreling, B., Caicedo, L., Mandelblatt, J., & Graves, K. D. (2011). Quality of life among immigrant Latina breast cancer survivors: realities of culture and enhancing cancer care. *Journal of Cancer Education*, *26*(4), 724-733. (33)	Y	Y	Y	Y	Y	Y	N	Y	Y	Y	9
Lopez-Class, M., Gomez- Duarte, J., Graves, K., & Ashing-Giwa, K. (2012). A contextual approach to understanding breast cancer survivorship among Latinas. Psycho-Oncology, 21(2), 115-124. (34)	U	Y	Y	Y	Y	Y	U	Y	Y	Y	8
Haq, R., Heus, L., Baker, N. A., Dastur, D., Leung, F. H., Leung, E., ... & Parsons, J. A. (2013). Designing a multifaceted survivorship care plan to meet the information and communication needs of breast cancer patients and their family physicians: results of a qualitative pilot study. *BMC medical informatics and decision making*, *13*(1), 1-13. Canada (24)	Y	Y	Y	Y	Y	Y	Y	Y	N	Y	9
Hill-Kayser, C. E., Vachani, C. C., Hampshire, M. K., Di Lullo, G., Jacobs, L. A., & Metz, J. M. (2013). Impact of internet-based cancer survivorship care plans on health care and lifestyle behaviors. *Cancer*, *119*(21), 3854-3860. (32)	Y	Y	Y	Y	Y	Y	N	N	Y	Y	8
Juarez, G., Mayorga, L., Hurria, A., & Ferrell, B. (2013). Survivorship education for Latina breast cancer survivors: empowering survivors through education. *Psicooncologia*, *10*(1), 57. DOI: 10.5209/rev_PSIC. 2013.v10.41947. USA (27)	Y	Y	Y	Y	Y	Y	Y	Y	Y	U	9
Singh–Carlson, S., Wong, F., Martin, L., & Nguyen, S. K. A. (2013). Breast cancer survivorship and South Asian women: understanding about the follow-up care plan and perspectives and preferences for information post treatment. *Current Oncology*, *20*(2), 63-79. (35)	Y	Y	N	Y	Y	Y	Y	Y	Y	Y	9
Wen, K. Y., Fang, C. Y., & Ma, G. X. (2014). Breast cancer experience and survivorship among Asian Americans: a systematic review. *Journal of Cancer Survivorship*, *8*, 94-107. (19)	Y	Y	Y	Y	N	Y	Y	Y	Y	Y	9

Crawford, J., Frisina, A., Hack, T., & Parascandalo, F. (2015). A peer health educator program for breast cancer screening promotion: Arabic, Chinese, South Asian, and Vietnamese immigrant women's perspectives. *Nursing Research and Practice*, *2015. (18)*	Y	Y	Y	Y	Y	U	Y	Y	Y	Y	9
Ahmed, K., Marchand, E., Williams, V., Coscarelli, A., & Ganz, P. A. (2016).Development and pilot testing of a psychosocial intervention program for young breast cancer survivors. *Patient education and counseling*, *99*(3), 414-420. (38)	Y	U	Y	Y	U	Y	U	Y	Y	Y	7
Burke, N. J., Napoles, T. M., Banks, P. J., Orenstein, F. S., Luce, J. A., & Joseph, G. (2016). Survivorship care plan information needs: perspectives of safety-net breast cancer patients. *PloS one*, *11*(12), e0168383. doi:10.1371/journal.pone.0168383. United states of America. (22)	Y	Y	Y	U	Y	U	Y	U	Y	Y	7
Gripsrud, B. H., Brassil, K. J., Summers, B., Søiland, H., Kronowitz, S., & Lode, K. (2016). Capturing the experience: Reflections of women with breast cancer engaged in an expressive writing intervention. *Cancer Nursing*, *39*(4), E51. (29)	Y	Y	Y	Y	Y	Y	Y	N	Y	Y	9
Tompkins C, Scanlon K, Scott E, Ream E, Harding S, Armes J. Survivorship care and support following treatment for breast cancer: a multi-ethnic comparative qualitative study of women's experiences. BMC health services research. 2016 Dec 1;16(1):401. (36)	Y	U	Y	Y	Y	Y	Y	Y	Y	Y	9
Warmoth, K., Cheung, B., You, J., Yeung, N. C., & Lu, Q. (2017). Exploring the social needs and challenges of Chinese American immigrant breast cancer survivors: a qualitative study using an expressive writing approach. *International journal of behavioral medicine*, *24*, 827-835. (40)	Y	Y	U	Y	Y	Y	Y	Y	Y	Y	9
Addington, E. L., Sohl, S. J., Tooze, J. A., & Danhauer, S. C. (2018). Convenient and Live Movement (CALM) for women undergoing breast cancer treatment: Challenges and recommendations for internet-based yoga research. *Complementary therapies in medicine*, *37*, 77-79. (37)	U	Y	Y	Y	Y	U	U	Y	Y	Y	7
Green, E. K., Wodajo, A., Yang, Y., Sleven, M., & Pieters, H. C. (2018). Perceptions of support groups among older breast cancer survivors: "I've heard of them, but I've never felt the need to go". *Cancer nursing*, *41*(6), E1. doi:10.1097/NCC.0000000000000522. USA. (25)	Y	Y	Y	Y	Y	Y	N	Y	Y	U	8
Nápoles, A. M., Santoyo-Olsson, J., Chacón, L., Stewart, A. L., Dixit, N., & Ortiz, C. (2019). Feasibility of a mobile phone app and telephone coaching survivorship care planning program among Spanish-speaking breast cancer survivors. *JMIR cancer*, *5*(2), e13543. (28)	Y	Y	Y	Y	U	Y	Y	Y	Y	Y	9
Brennan, L., Kessie, T., & Caulfield, B. (2020). Patient experiences of rehabilitation and the potential for an mHealth system with biofeedback after breast cancer surgery: qualitative study. *JMIR mHealth and uHealth*, *8*(7), e19721. (21)	Y	U	Y	Y	Y	Y	Y	Y	Y	U	8

Levesque, J. V., Gerges, M., & Girgis, A. (2020). Psychosocial experiences, challenges, and coping strategies of Chinese–Australian women with breast cancer. *Asia-Pacific Journal of Oncology Nursing*, *7*(2), 141-150. (26)	Y	Y	Y	Y	Y	Y	Y	Y	N	Y	9
Costas-Muñiz, R., Garduño-Ortega, O., Hunter-Hernández, M., Morales, J., Castro-Figueroa, E. M., & Gany, F. (2021). Barriers to psychosocial services use for Latina versus non-Latina white breast cancer survivors. *American journal of psychotherapy*, *74*(1), 13-21. (31)	Y	Y	Y	Y	U	Y	Y	Y	Y	Y	9
Total ratings for 22 articles	20	19	19	21	18	18	16	18	20	19	188
Total percentage of 22 articles	91%	86%	86%	95%	82%	82%	73%	82%	91%	86%	85%

Y = Yes, N = No, U = Unclear; JBI Critical Appraisal Checklist for Qualitative Research. Q1 = Is there congruity between the stated philosophical perspective and the research methodology? Q2 = Is there congruity between the research methodology and the research question or objectives? Q3 Is there congruity between the research methodology and the methods used to collect data? Q4 = Is there congruity between the research methodology and the representation and analysis of data? Q5 = Were those delivering treatment blind to treatment assignment? Q6 = Is there a statement locating the researcher culturally or theoretically? Q7 = Is the influence of the researcher on the research, and vice-versa, addressed? Q8 = Are participants, and their voices, adequately represented? Q9 = Is the research ethical according to current criteria or, for recent studies, is there evidence of ethical approval by an appropriate body? Q10 = Do the conclusions drawn in the research report flow from the analysis, or interpretation, of the data? Scoring for each article: 7-10 High, 4-6 Medium, 1-3 Low impact.

Table S2. JBI Critical Appraisal Checklist for Qualitative Systematic Reviews

Reviewer Date
Author Year Record Number

	Yes	No	Unclear	Not applicable
1. Is the review question clearly and explicitly stated?	□	□	□	□
2. Were the inclusion criteria appropriate for the review question?	□	□	□	□
3. Was the search strategy appropriate?	□	□	□	□
4. Were the sources and resources used to search for studies adequate?	□	□	□	□
5. Were the criteria for appraising studies [illegible]	□	□	□	□
6. Was critical appraisal conducted by two or more reviewers independently?	□	□	□	□
7. Were there methods to minimize errors in data extraction?	□	□	□	□
8. Were the methods used to combine studies [illegible]	□	□	□	□
9. Was the likelihood of publication bias assessed?	□	□	□	□
10. Were recommendations for policy and/or practice supported by the reported data?	□	□	□	□
11. Were the specific directives for new research appropriate?	□	□	□	□

Overall appraisal: Include □ Exclude □ Seek further info □
Comments (Including reason for exclusion)

Table S3. Data extraction instrument

Reference, title, authors	Methods, design, approach	Phenomena, intervention	Population, Comparison	Outcomes	Results, Conclusion
Addington, E. L., Sohl, S. J., Tooze, J. A., & Danhauer, S. C. (2018). Convenient and Live Movement (CALM) for women undergoing breast cancer treatment: Challenges and recommendations for internet-based yoga research. *Complementary therapies in medicine, 37*, 77-79.	Pilot trial of cancer-adapted yoga classes delivered via internet-based, multipoint videoconferencing to women undergoing radiation or chemotherapy for breast cancer. Recruited women who met the inclusion criteria (e.g., stage 0-III breast (GoToMeeting). The participants were asked to attend 12 biweekly, 75-minute, Integral yoga classes that included gentle postures, breathing,	Feasibility and acceptability of delivering cancer-adapted yoga classes via internet-based videoconferencing to women undergoing radiation or chemotherapy for breast cancer. Interested in whether this approach could overcome the barriers yoga participants faced by many cancer patients, such as physical limitations, fatigue, transportation, issues, and scheduling conflicts. They also wanted to explore how the participants and the yoga	Women who were undergoing radiation or chemotherapy for breast cancer. The participants met the inclusion criteria, which included having stage 0-III breast cancer and elevated distress. The participants did not meet the exclusion criteria, which included regular yoga or vigorous exercise, and recent or planned surgery. The participants were recruited from a comprehensive cancer centre in the southeastern US. The participants were provided with internet-connected computers	Difficulties in feasibility and accessibility for the program Recruitment: Difficulties in recruiting participants who met the inclusion and exclusion criteria. Retention: Challenges in retaining participants throughout the study, mainly due to cancer-related barriers. Adherence: Collected data on the participant's adherence to the online yoga program, including attendance at the yoga classes. Participant Focused Feedback & Acceptability Satisfaction: Measured the participants' satisfaction with the online yoga program using	Feasibility and acceptability of online yoga classes for women undergoing treatment for breast cancer. Despite recruitment and retention challenges due to cancer-related and technological barriers, the program was generally well-received. Improvements suggested including more flexible class times and enrolling.

	meditation, and relaxation. The classes were taught by a Registered Yoga Teacher with specialized training in cancer-adapted yoga. The participants could see and interact with the instructor and other participants during the classes. Collected data on feasibility and acceptability, including enrollment rate, retention, adherence, satisfaction ratings, and qualitative feedback from program evaluation forms and telephone interviews.	instructor perceived the online yoga program and how it could be improved.	and detailed instructions on how to install and use the videoconferencing software. The participants were asked to attend 12 biweekly, 75-minute, integral yoga classes that included gentle postures, breathing, meditation, and relaxation. The classes were taught by a Registered Yoga Teacher with specialized training in cancer-adapted yoga. The participants could see and interact with the instructor and other participants during the classes.	satisfaction ratings. Qualitative feedback: Qualitative feedback from the participants and the yoga instructor on how to improve the online yoga program. Improvement Class times: Offering more varied class times to enhance the feasibility and acceptability of the online yoga program. Enrollment timing: Recommended enrolling patients after they complete treatment to minimize cancer-related barriers. Technology interface: Simplifying the technology interface to make it easier for participants to use.	

Ahmed, K., Marchand, E., Williams, V., Coscarelli, A., & Ganz, P. A. (2016). Development and pilot testing of a psychosocial intervention program for young breast cancer survivors. *Patient education and counseling*, *99*(3), 414-420.	Needs assessment with community organizations and YBCS to identify the gaps in services and the priorities for the intervention. They also involved advisory committees composed of survivors and community representatives in the program planning process. Psychosocial program based on evidence-based models of treatment that included psychoeducation, skill-building exercises, and discussion. The program	The psychosocial challenges faced by young breast cancer survivors (YBCS). These challenges include managing anxiety, fear of recurrence, decision-making, and coping with sexuality/relations issues.	The participants were young breast cancer survivors (YBCS) who were diagnosed before the age of 45 and who had completed active treatment, excluding hormonal therapy. The participants were recruited from local community organizations, medical facilities, and social media in the Los Angeles area3. The workshop group sizes ranged from 3 to 8 participants. Only limited demographic data were available for participants from UCLA, where the mean age was 41.7 years (median 43 years)	Psychosocial challenges of Young Breast Cancer Survivors (YBCS): explores the unique psychosocial issues that YBCS face, such as anxiety, fear of recurrence, decision-making difficulties, and sexuality/relationship issues. Development and effectiveness of a psychosocial intervention program: Creation of an intervention program designed to address the psychosocial challenges identified in the first theme. It covers the process of needs assessment, stakeholder engagement, and pilot testing. Effectiveness - discusses	Presents a psychosocial intervention program developed for young breast cancer survivors (YBCS) to address their unique challenges such as anxiety, fear of recurrence, decision-making, and sexuality/relationship issues. The program was evaluated using pre-and post-test questionnaires and qualitative feedback surveys, and the results showed significant improvements in managing anxiety and improving relationships/sexuality issues. Program fills an unmet need for YBCS and provides a model for integrating psychosocial care with oncology treatment. Wider

	had two modules: one focused on anxiety management and the other on relationships and sexuality. The program was delivered in a group format with a workbook for each participant. Impact of the intervention on the participants' knowledge, confidence, and ability regarding key concepts using a questionnaire before and after the program. They also collected qualitative feedback from the participants. The results showed an increase in self-reported scores on			the evaluation of the intervention program's effectiveness in improving psychosocial outcomes for YBCS. It includes the analysis of pre-and post-test questionnaires and qualitative feedback surveys. Dissemination and Integration of the Intervention Program: This theme looks at how the intervention program was disseminated to community clinical settings and integrated into oncology care. It discusses the training of local facilitators and the potential for broader implementation.	dissemination of the program and further research for its continuous improvement.

	both modules and positive comments on the program content and format.				
Ashing-Giwa, K. T., Padilla, G. V., Tejero, J. S., & Kim, J. (2004). Breast cancer survivorship in a multiethnic sample: challenges in recruitment and measurement. *Cancer: Interdisciplinary International Journal of the American Cancer Society*, *101*(3), 450-465.	Qualitative research methods to gain a deep understanding of the experiences and concerns of breast cancer survivors from four ethnic groups: African American, Asian American, Latina, and Caucasian. Data were collected through key informant interviews with 20 health professionals and focus group interviews with 102 breast cancer survivors.	Personal experiences of breast cancer survivors from four different ethnic backgrounds: African American, Asian American, Latina, and Caucasian. It aims to shed light on their journeys from the point of diagnosis, through the treatment process, and into their lives post-cancer. The study recognizes that each ethnic group may face unique challenges and have distinct perspectives based on their cultural and societal contexts. For instance, language	Ethnicity and socioeconomic background: The study involves breast cancer survivors from four different ethnic backgrounds: African American, Asian American, Latina, and Caucasian. These women have varying socioeconomic and cultural contexts that may influence their quality of life and psychosocial experiences. Health-related quality of life: The study assesses the physical, functional, psychological/emotional, and social well-being of breast cancer survivors about their	Culture and breast cancer experiences: Explores how culture influences the breast cancer experience of women from different ethnic groups, such as African American, Asian American, Latina and Caucasian. Culture affects women's health beliefs, coping strategies, family support, spirituality, and access to care. Discusses the need for culturally responsive healthcare services and programs. Psychosocial functioning and social support: examines the psychosocial	Diverse Experiences: The study found that breast cancer survivors from different ethnic backgrounds (African American, Asian American, Latina, and Caucasian) have unique experiences. These experiences are shaped by their cultural and societal contexts. For instance, cultural beliefs about illness and health can influence how these women perceive their diagnosis and treatment. Their societal context, including factors like healthcare access and social support, can also impact

	The key informant interviews provided insights into the professional perspective on the needs and concerns of breast cancer survivors, while the focus group interviews allowed for first-hand accounts of the survivors' experiences and concerns. The research design is cross-sectional and comparative, collecting data at one point in time and comparing the experiences and concerns of breast cancer survivors across different	barriers, healthcare access, and social support systems can vary greatly among these groups. These factors can significantly impact a survivor's experience with breast cancer. By exploring these diverse experiences, the study seeks to inform healthcare providers and policymakers about the specific needs of different ethnic groups. This could lead to the development of more tailored support systems and healthcare interventions that take into account cultural sensitivities and disparities. This underscore the importance of diversity in healthcare	health. It explores the unique challenges and perspectives of different ethnic groups. Cultural and socio-ecological factors: The study examines how culture and socio-ecological factors impact the breast cancer experience of women from diverse backgrounds. These factors include health beliefs, health socialization, relationships, quality of care, spirituality, and work issues.	impact of breast cancer on women's quality of life, emotional well-being, and mental health. Identifies common psychological issues such as fear, anxiety, denial, and depression. Describes how women use various social resources such as family, friends, support groups, and health professionals to deal with breast cancer. Identity and role changes: how breast cancer affects women's sense of identity, such as their roles as mothers, wives, and caregivers. Highlights how women's identity is shaped by their ethnicity, acculturation, socioeconomic status, and education. How women cope with	their journey through breast cancer. offer more effective support and interventions. Policy Implications: Significant implications for healthcare policies. It suggested that policies need to address healthcare disparities and provide adequate support for cancer survivors from diverse backgrounds. This could involve improving access to healthcare resources, enhancing patient education about the disease, or developing support programs tailored to the needs of different ethnic groups. Understanding these diverse experiences can help healthcare providers offer more

	ethnic groups. Socio-ecological approach, recognizing that individuals are embedded within larger social systems that can influence health outcomes. This comprehensive approach allows for a thorough exploration of the unique challenges faced by breast cancer survivors from diverse ethnic backgrounds.	research. It highlights that a one-size-fits-all approach may not be effective in addressing health issues as complex as cancer. Instead, a more nuanced understanding of patients' experiences across different ethnic backgrounds can lead to more inclusive and effective healthcare solutions.		identity changes and challenges due to breast cancer. Barriers and facilitators of health care and psychosocial care evaluates the barriers and facilitators to accessing health care and psychosocial care among women with breast cancer, especially women of color. Emphasizes the importance of socioeconomic and cultural factors, as well as systemic problems in the health care system. Benefits of health education, patient empowerment, and cultural competence.	personalized care. Healthcare Disparities: Disparities in healthcare access and quality among different ethnic groups. These disparities can be due to a variety of factors, such as language barriers, socioeconomic status, and geographical location. For example, women who do not speak English as their first language might face challenges in understanding their diagnosis or treatment plan.
Brennan, L., Kessie, T., & Caulfield, B. (2020). patient experiences of rehabilitation and the potential for an mHealth	Qualitative research study design to explore the experiences and needs of women	Breast cancer rehabilitation: physical and psychological challenges that women face after breast cancer surgery and how physiotherapy	The study included 10 women who had surgery for breast cancer within the last 5 years. They were all white Irish, with a range of	Rehabilitation experiences: patients' rehabilitation process, their awareness of the impact of surgery, their expectations and reality of physiotherapy	Main themes from the interviews with 10 breast cancer survivors: acute and long-term consequence

system with biofeedback after breast cancer surgery: qualitative study. *JMIR mHealth and uHealth*, *8*(7), e19721.	during home rehabilitation following surgery for breast cancer and to gather survivors' perspectives on and requirements from mHealth technology for postoperative breast cancer rehabilitation. Semi structured interviews: Semi structured interviews with 10 breast cancer survivors under two main topics: "Rehabilitation" and "Technology". The interviews were audio-recorded, transcribed, and anonymized. Thematic	can help them recover and prevent upper limb dysfunction. Unmet needs and lack of support: Gaps in the current provision of physiotherapy services and information for breast cancer survivors and how they affect their rehabilitation outcomes and quality of life. mHealth technology investigates the potential of mobile health (mHealth) systems, such as apps and wearable sensors, to enhance breast cancer rehabilitation by providing exercise support, biofeedback, and information. User-centered design: TUser-centered design approach to understand the experiences and needs of breast cancer survivors and	education levels, employment statuses, and incomes. They were mostly married or in a domestic partnership, and their ages ranged from 35 to 74 years. The participants had different types of breast surgery, such as lumpectomy, mastectomy, and reconstruction, and different extents of axillary surgery, such as sentinel lymph node biopsy or axillary clearance. They also received different types of postoperative physiotherapy, from a single assessment to follow-up appointments or referrals. The participants were recruited from a sports club for breast cancer survivors, which may indicate that they were	care, their confidence, and challenges in doing home exercises, and their sources of information and support. Unmet needs and lack of support: Gaps in physiotherapy services, information provision, and emotional support for patients after breast cancer surgery. It also highlights the patients' feelings of isolation, fear, and anxiety during home rehabilitation. Self-driven rehabilitation: Describes the patients' motivation, proactivity, and resilience in pursuing their recovery. It also discusses the benefits of exercise, peer support, and digital	s of surgery, unmet needs and lack of support, self-driven rehabilitation, and visions for high-quality rehabilitation. These themes reflected the participants' experiences, challenges, and preferences during home rehabilitation after breast cancer surgery. Unmet needs and mHealth potential: Participants had unmet needs regarding information, physiotherapy, and support during home rehabilitation, and they felt isolated and uncertain about their recovery. mHealth system could address these unmet needs by

	analysis: Thematic analysis with a semantic, mixed inductive and deductive approach to analyze the interview data, following the process outlined by Braun and Clarke. Identified four main themes under the topic of "rehabilitation experiences" and two main themes under the topic of "technology". Reported the findings according to the Consolidated Criteria for Reporting Qualitative Research (COREQ) checklist for qualitative studies.	their preferences and requirements for an mHealth system.	more physically active than the general population of breast cancer patients	technology for enhancing physical and psychological well-being. Visions for high-quality rehabilitation: Patients' preferences and recommendations for improving breast cancer rehabilitation. These include more access to physiotherapy, more patient education, more personalized and positive content, and more feedback and encouragement. mHealth system: Participants were open to using an mHealth system for postoperative rehabilitation and expressed their requirements for such a system.	providing exercise support, biofeedback, and information from a reliable source. Motivation and quality of rehabilitation: Participants were motivated to exercise and recover after surgery, but faced barriers such as treatment side effects, fear of movement, and lack of feedback. mHealth system could improve the quality of rehabilitation by providing visual and audio guidance, personalized and positive content, and remote monitoring by a physiotherapist. User requirements for an
Burg, M. A., Lopez, E. D.,	Qualitative research:	Survivorship care plans	Female breast	Minority breast	Information gaps: The

Dailey, A., Keller, M. E., & Prendergast, B. (2009). The potential of survivorship care plans in primary care follow-up of minority breast cancer patients. *Journal of General Internal Medicine, 24*(2), 467. DOI: 10.1007/s11606-009-1012-y. United States of America.	Qualitative methods to explore the perspectives of minority breast cancer survivors on their follow-up care and survivorship care plans. Qualitative methods are useful for understanding the experiences, meanings, and contexts of people's lives. Focus groups: Four focus groups with 32 minority breast cancer survivors living in a Southeastern urban area. Focus groups are a form of group interview that allows participants to interact with each other and	(SCPs): These are documents that provide information on the cancer diagnosis, treatment, follow-up care, and psychosocial support for cancer survivors and their primary care providers. Explores the potential benefits and challenges of SCPs for minority breast cancer survivors. Minority breast cancer survivors' experiences reports the findings of four focus groups with minority breast cancer survivors, mostly African American women, who shared their recall of information from their oncologists, their unmet needs and concerns, and their opinions on the ASCO	cancer survivors: All the participants were women who had completed active treatment for breast cancer and were between the ages of 18 and 65. Minority and mainly African American: Three out of four focus groups comprised only African American women; the fourth group included six African American women, one Hispanic woman and two Caucasian women. Living in a Southeastern urban area: The participants were recruited from members of the Sisters Network (a national African American organization	cancer survivors' information needs: Survivors often have information gaps, confusion, and dissatisfaction with the communication from their oncologists and primary care providers. Minority-specific considerations, such as race-related side effects and support resources, should be taken into account when designing and delivering information to this population. Survivorship care plans as a potential tool for improving information transfer and coordination of care examines the concept and content of survivorship care plans	survivors felt that they did not receive enough information from their oncologists about their cancer diagnosis, treatment, side effects, and follow-up care. They often had to seek information from other sources, such as the Internet, support groups, and other survivors. Psychosocial distress: The survivors experienced various physical and psychological side effects that were not anticipated or addressed by their oncologists. They also felt anxious and abandoned after completing treatment and wished they had more guidance on how to cope and prevent

	share their views on a topic. Focus groups can generate rich and diverse data on people's opinions, attitudes, and feelings. Purposive sampling: Purposive sampling to recruit eligible participants who were female breast cancer survivors between the ages of 18 and 65 whose active treatment for breast cancer was completed. Purposive sampling is a technique that selects participants based on specific criteria or characteristics that are relevant to the research question. Purposive sampling	SCP template for breast cancer. Primary care follow-up of cancer survivors: Discusses the implications of SCPs for improving the coordination and quality of care for cancer survivors in primary care settings, especially for minority and underserved populations. Identifies the gaps in knowledge and training for primary care physicians who care for cancer survivors.	with regional breast cancer support groups) and urban public health department outpatient clinics serving a high proportion of minority patients in a Southeastern city.	(SCPs), which are portable records of tumour characteristics, treatments received, and follow-up guidelines meant to be completed by the oncologist and given to the survivor and their primary care provider. Assesses the reactions and opinions of minority breast cancer survivors on the SCP template from the Association of Clinical Oncology (ASCO) and identifies the strengths and limitations of the SCP as a quality improvement tool. Discusses the system-level and provider-level challenges and opportunities for implementing SCPs in clinical practice.	recurrence. Survivorship care plan feedback: The survivors liked the idea of having an SCP, but found the ASCO template too technical, generic, and limited. They suggested that the SCP should be more personalized, use plain language, and include more information on side effects, self-care, and resources.

	can enhance the validity and representativeness of the sample.				
Burke, N. J., Napoles, T. M., Banks, P. J., Orenstein, F. S., Luce, J. A., & Joseph, G. (2016). Survivorship care plan information needs perspectives of safety-net breast cancer patients. *PloS one*, *11*(12), e0168383. doi:10.1371/journal.pone.0 16 8383. United States of America.	Community-university collaboration between the San Francisco Women's Cancer Network (SFWCN) and the University of California, San Francisco (UCSF). The collaboration aimed to identify the informational and structural challenges to treatment and survivorship for safety-net breast cancer patients, and to inform the content and delivery of	Survivorship care plans: SCPs are written documents that summarize the care received, disease characteristics, and follow-up plans for cancer survivors. Discusses the challenges and gaps in implementing SCPs for diverse and underserved populations and the recommendations from the Institute of Medicine (IOM) and other organizations. Safety-net breast cancer patients: These are patients who receive care from providers who offer services regardless of ability to pay,	The study engaged 38 participants with diverse characteristics to explore the information needs of safety-net breast cancer patients regarding survivorship care plans (SCPs). The participants, with a mean age of 61, represented various racial/ethnic backgrounds and educational levels. Notably, 36% reported an annual household income below $10,000. The findings emphasized a need for clear communication on the transition from active treatment to survivorship,	Need for information and education on the transition to survivorship: Women in the focus groups expressed confusion and a lack of clarity regarding the transition from "active treatment" to "survivorship." Despite being on ongoing treatments like Tamoxifen or other hormonal therapies, women found themselves categorized as in survivorship, leading to a discrepancy in understanding between patients and healthcare	Need for Information and Education on the Transition to Survivorship: Breast cancer survivors expressed confusion and a lack of clarity about the transition from active treatment to survivorship. The ongoing nature of treatments, such as hormonal therapies, created uncertainty about the categorization of survivorship. Participants suggested the need for new terms and highlighted the role of community organizations and support

	appropriate and useful survivorship care plans (SCPs). Qualitative methods, specifically focus groups, to elicit the perspectives of low literacy, multi-lingual breast cancer patients on their information needs and preferences for SCPs. The focus groups were conducted in five languages (English, Spanish, Cantonese, Russian, and Tagalog) with women who were at least two years and no more than 15 years from their diagnosis.	and who have low health literacy, low English proficiency, and low income. Reports the findings from six focus groups conducted in five languages with African American, Latina, Russian, Filipina, White, and Chinese breast cancer patients who are at least two years and no more than 15 years from their diagnosis. Information needs and preferences: Identifies three themes that reflect the information needs and preferences of the focus group participants: 1) the need for information and education on the transition between "active treatment" and "survivorshi	highlighting confusion among participants. The study sheds light on structural issues within the healthcare context, such as fleeting relationships with providers and poor communication between oncology and primary care, underlying the reported information needs and care experiences.	providers. Participants who did have an understanding of survivorship often gained this knowledge from community organizations and support groups rather than their healthcare providers. Information needed from providers Participants highlighted various areas where they lacked information from healthcare providers, contributing to a sense of uncertainty and anxiety. Five primary areas of information deficit were identified: Screening and Monitoring: Participants were unsure about the frequency and types of screenings they should undergo during	groups in providing information on survivorship. Information Needed (and Often Not Obtained) from Providers: Participants reported information deficits in key areas: Screening and Monitoring: Uncertainty about the frequency and types of screenings during survivorship. Recurrence: Fears and uncertainties about cancer recurrence, seeking guidance on symptoms and reactions. Side Effects and Pain: Lack of truthful information from providers, especially regarding hormonal treatments and prevalent side effects like

	The focus groups were facilitated by bilingual, bicultural community organization staff who had undergone a half-day training with the university partners. Standard qualitative analysis techniques, including iterative data review, multiple coders, and "member checking".	p". 2) information needed (and often not obtained) from providers, such as screening, recurrence, side effects, reconstruction, and healthy lifestyle; and 3) perspectives on SCP content and delivery, such as referrals, symptom management, hereditary risk, family communication, and self-care strategies.		survivorship. Recurrence: Women expressed fears and uncertainties about cancer recurrence, seeking guidance on recognizing symptoms and how to react. Healthy Lifestyle Choices: Participants desired guidance on healthy eating, physical activity, and managing the effects of cancer on their daily lives.	lymphedema. Reconstruction: Insufficient information on breast reconstruction alternatives, timing, and post-surgery care. Healthy Lifestyle Choices: Desire for guidance on healthy eating, physical activity, and managing the effects of cancer on daily life. Perspectives on SCP Content and Delivery: Participants expressed specific
Costas-Muñiz, R., Garduño Ortega, O., Hunter Hernández, M., Morales, J., Castro-Figueroa, E. M., & Gany, F. (2021). Barriers to psychosocial services use for Latina versus non-	Retrospective cross-sectional design and was conducted at a single comprehensive cancer center located in New York City (NYC). Participant	Access and utilization of psychosocial services among breast cancer survivors, with a specific emphasis on understanding the barriers faced by Latina and	Adult breast cancer survivors aged 21 or older, in remission from their diagnosis, and received care at a comprehensive cancer center in New York City between 2009 and 2014. The	Disparities in Psychosocial Outcomes: The study delves into the disparities in psychosocial outcomes, specifically examining how Latina individuals living with breast cancer experience poorer quality	Ethnic Disparities in Psychosocial Service Barriers: Latinas, among those needing psychosocial services, face specific barriers.

Latina white breast cancer survivors. *American journal of psychotherapy*, *74*(1), 13- 21.	s: The study included adult survivors aged 21 or older in remission from breast cancer. The sample consisted of 409 Latinas and 5146 non-Latina Whites who had received cancer care at the center between 2009 and 2014. The selection involved including all 409 Latinas and a random 10% sample of non-Latina Whites. Data Collection: Medical records of individuals treated between 2009 and 2014 were reviewed. Eligible participants received a mailed questionn	non-Latina White individuals. The study aims to explore disparities in psychosocial outcomes, investigate the reasons behind the lower rates of psychosocial service utilization among Latinos, and examine the self-reported barriers to accessing psychosocial services among breast cancer survivors. Additionally, the study explores the associations between these barriers and the actual use of psychosocial services following a cancer diagnosis.	sample consists of 409 Latina and 5146 non-Latina White individuals. The selection method involved including all Latina participants and a random 10% sample of non-Latina Whites, totaling 409 and 5146, respectively. The study explores a range of demographic characteristics, including marital status, socioeconomic status, family composition, living situation, employment status, ethnicity, race, language preference, birthplace, and years of living in the United States. It also considers medical factors such as the time since diagnosis	of life and higher rates of depression compared to non-Latino Whites. Utilization of Psychosocial Services: The research investigates the patterns of access to psychosocial services among breast cancer survivors. It explores the utilization rates of psychological, psychiatric, and other psychosocial interventions, with a particular focus on the differences between Latina and non-Latina White individuals. Barriers to psychosocial services use: The study aims to identify and understand the barriers that hinder the use of psychosocial services, especially among Latina breast cancer survivors. It acknowledges	Barriers include language, cultural understanding, and perceived cost. Prior Study on Psychosocial Service Disparities: Previous research showed differences in availability and acceptability. Non-Latina Whites are more likely to have contact with social workers and receive psychotropic medication. Latinas are more likely to receive spiritual counselling. Commonly Cited Barriers to Psychosocial Care: Beliefs in self-reliance, a desire to return to normalcy, and reliance on family and friends. Key reasons survivors decline

	aire packet covering demographics, attitudes toward and history of using psychosocial services, and perceived barriers to use. Implied consent was obtained through the completion of the anonymous survey. Demographic variables collected included age, education, religion, marital status, socioeconomic status, family composition, living situation, employment status, ethnicity, race, language preference, birthplace, and years of living in		and remission status. Due to a small sample size, separate analyses for Latina and non-Latina White subgroups were not conducted. The response rate for the study was 30%, and participants provided implied consent by completing an anonymous survey after reviewing a form detailing the study, their rights, and potential benefits. These participant characteristics offer a comprehensive view of the study population, crucial for interpreting the findings on psychosocial outcomes and barriers to accessing psychosocial services among breast cancer survivors.	that despite the need for mental health support, there are obstacles preventing individuals, particularly Latinos, from receiving appropriate services. Unique Concerns of Latino Individuals: Latinos often face unique challenges, including socioeconomic, cultural, and linguistic issues, impacting their access to psychosocial and mental health services. The study aims to uncover and comprehend these unique	services. Predictors of Lack of Psychosocial Service Use: Desire to return to normalcy. Lack of understanding about therapy benefits. Not knowing where to receive services. Mental health stigma and beliefs about distress normalcy. Concerns about cultural understanding by counsellors. Discomfort with medical interpreters. The gap in National Clinical Guidelines: Lack of specialized recommendations for culturally diverse patients. Need for guidelines addressing distress screening and management for diverse populations. Importance

	the United States. Psychosocial services variables encompassed histories of usage, preferences for services, and types of mental health professionals involved. Barriers variables included 14 patient-related barriers and six physician- or system-related barriers. Data Analysis: SPSS 19 software was utilized for statistical analyses.			concerns that may contribute to disparities in mental health outcomes. Financial Impact of Cancer Diagnosis: The study explores the association between a cancer diagnosis, worsened mental health, and its impact on income, particularly emphasizing the challenges faced by Latinos in comparison to non-Latino Whites. Psychosocial Service Use Disparities: The study highlights the disparities in the use of psychosocial services, revealing that Latinos are less likely to receive these services than non-Latino Whites. It explores the	of Educational Interventions: Need for educational programs at individual, provider, and institutional levels. Programs to increase awareness, normalize psychosocial service use, and provide information. Emphasis on cultural competency training for providers. Call for System-Level Interventions: Recommendations for institutional policies promoting cultural competency. Support for diverse, bilingual staff. Translation of materials and language assistance programs.

				potential reasons for this gap, encompassing patient-, provider-, and system-related barriers.	
Crawford, J., Frisina, A., Hack, T., & Parascandalo, F. (2015). A peer health educator program for breast cancer screening promotion: Arabic, Chinese, South Asian, and Vietnamese immigrant women's perspectives. *Nursing Research and Practice, 2015.*	Participatory action research (PAR): This is a collaborative and reflective approach that involves the participants as co-researchers in the study. The researchers used PAR to explore the experiences of immigrant women with a peer health educator program that promoted breast health and screening. The researchers drew on the critical social	Immigrant women's experiences with a peer health educator program: The main topic of interest is how immigrant women perceived and valued the program, which aimed to reduce barriers and provide access to breast health information and mammography screening. The researchers explored the benefits and challenges of the program, as well as the strengths and areas of improvement. Breast cancer prevention and screening: A related topic of interest is how immigrant women learned about	Reports on a study that involved 82 immigrant women from four different cultural groups: Arabic, Chinese, South Asian, and Vietnamese. The participants were recruited from a peer health educator program that provided access to breast health information and mammography screening. The participants were 40 years of age and older, and had attended a women's health session or been accompanied to breast screening by a peer health educator. The majority of	Identity and acculturation: The study explores how immigrant women from different cultural backgrounds adapt to their new host country and how they perceive their health and well-being. The study also examines how the peer health educator program helps immigrant women develop a sense of identity and belonging in Canada. Culture and health literacy: The study highlights the importance of cultural competence and health literacy in promoting breast cancer prevention	Immigrant women's experiences with the peer health educator program: Explored how Arabic, Chinese, South Asian, and Vietnamese immigrant women in Canada perceived a public health program that used peer health educators and multiple strategies to promote breast health and mammography screening. Participatory action research and qualitative methods to collect and analyze data from 82 immigrant women who participated in focus groups and interviews.

	theory of Freire and Fals Borda to guide their PAR process. Qualitative methods: The researchers used focus groups and one-on-one interviews to collect data from 82 immigrant women from four cultural groups: Arabic, Chinese, South Asian, and Vietnamese. The researchers used a semistructured interview guide with open-ended questions to elicit the perspectives of the participants. The focus groups and	breast health, risk reduction, breast cancer, and screening services through the program. The researchers examined how the program influenced immigrant women's knowledge, attitudes, beliefs, and behaviors regarding breast cancer prevention and screening. Public health actions and implications: Another related topic of interest is how the researchers used the findings from the study to inform and improve the public health program and practice. The researchers reported on the public health actions that were taken based on the recommendations of the immigrant women. They also discussed the implications	participants were Arabic and South Asian immigrant women. Two-thirds of participants were 50 years of age and older. The mean number of years in Canada was 11 years, with a range of less than one year to 36 years. Over 50% of women resided in Canada for 10 years or less. The majority of immigrant women preferred to communicate in their native language.	and screening among immigrant women. The study shows how the peer health educator program provides culturally appropriate and linguistically accessible information and support to immigrant women, and how this enhances their knowledge, attitudes, and behaviors regarding breast health. Psychosocial care and empowerment: The study demonstrates the role of peer health educators and social networks in providing psychosocial care and empowerment to immigrant women. The study reveals how the peer health educator program fosters a caring and trusting environment, where	Four dominant themes emerged from the data: Identified four main themes that reflected immigrant women's experiences with the program: (1) Breast Cancer Prevention, which focused on learning about breast health, risk reduction, and screening services; (2) Social Support, which centered on the attributes of the peer health educator and the support she provided, as well as the support from other women in the community; (3) Screening Services Access for Women, which highlighted immigrant women's perceptions of the program as a whole and its connections to other health

	interview s were conducted in the preferred language of the participan ts by immigran t women facilitator s who were native speakers of the respective language. Themati c content analysis : The research ers used this method to analyze the transcri bed and translat ed data.	of the study for public health policy, research, and education.		immigrant women can share their experiences, feelings, and concerns, and receive emotional and practical support. The study also shows how the peer health educator program empowers immigrant women to take charge of their own health and access healthcare services.	services, as well as the role of family physicians; and (4) Program Enhancement s, which suggested ways to improve the program in terms of content, process, and dissemination. Implications for public health practice and research: Discussed how the findings provided insights into the strategies used to promote breast health and mammograph y screening among immigrant women, as well as the barriers that persist, such as the lack of information and recommendati on from family physicians.
Green, E. K., Wodajo, A., Yang, Y., Sleven, M., &	Qualitative methodolo gy: Uses grounded theory, an inductive,	Support groups: Aims to describe how older breast cancer survivors	Age: women who were at least 65 years old when they	Cultural differences in support-seeking: Support-seeking	Support groups are underutilize d by older breast cancer survivors:

Pieters, H. C. (2018). Perceptions of support groups among older breast cancer survivors: "I've heard of them, but I've never felt the need to go". *Cancer Nursing*, *41*(6), E1	qualitative methodology informed by constructivism, to explore how breast cancer survivors 65 years and above perceived professionally-led, in-person support groups. Grounded theory is a method that aims to generate theory from data through systematic coding, constant comparison, and memo-writing. Constructivism is a perspective that assumes that reality is socially constructed, and multiple interpretations are possible Individual interviews: Data from individual, in-depth	perceived professionally-l ed, in-person support groups, and why they did or did not attend them. Support groups are a type of psychosocial intervention that can provide emotional, informational, and instrumental support to cancer survivors. Reviews the literature on the benefits and challenges of support groups, and the factors that influence participation in them. Anti-hormonal treatment: Focuses on the decision-making processes related to persistence with anti-hormonal treatment by older breast cancer survivors in the posttreatment phase.	were diagnosed with breast cancer. The average age of the participants was 71.9 years at diagnosis. Ethnicity: Reports that most participants self-identified as white (n=44), while the rest did not specify their ethnicity. Education: Reports that most participants were at least college graduates (n=38), while the rest had lower levels of education. Support: Most participants identified their husband (n=21) or daughter (n=17) as their main support person and spoke with friends daily (n=43). All participants lived independently either with a spouse (n=24) or alone (n=23). Cancer	literature suggests differences among individuals from different cultural backgrounds, and that their sample mostly included Caucasian women. This implies that culture may influence how older breast cancer survivors perceive and utilize support groups and that more research is needed to capture the diversity of experiences and preferences among different cultural groups. Identity as a cancer survivor: Discusses how participants resisted an identity as a cancer patient, and how they wanted to move on from the cancer experience and return to their life	Interviewed 54 women who were 65 years or older when they were diagnosed with early-stage breast cancer. Only two of them were attending a professionally-led, in-person support group at the time of the interview. Most of them had negative assumptions about support groups and did not see themselves as the type of person who would benefit from them. Support groups are perceived as a place for emotional support only: Most participants thought that support groups were designed to provide emotional support for women who did not have other sources of support or who had more advanced or

	interviews with 54 women who were at least 65 years old when they were diagnosed with loco-regional breast cancer and who initiated anti-hormonal treatment. The interviews were conducted by the senior author, an experienced qualitative interviewer, in a private place of the participant's choosing. The interviews lasted an average of 97.2 minutes and covered topics such as support groups, decision-making, and anti-hormonal treatment.	Anti-hormonal treatment is a type of oral medication that can reduce the risk of recurrence and mortality for hormone receptor-positive breast cancer. Examines the role of support groups and other sources of support in facilitating adherence to anti-hormonal treatment.	stage: Participants were diagnosed with loco-regional (Stage I, II or III) breast cancer, and initiated anti-hormonal treatment. Sample consisted of two equal groups: 27 women continuing with an anti-hormonal treatment and 27 women who had prematurely discontinued the medication within the 15 months preceding the interview.	before cancer. This suggests that attending a support group may challenge their sense of identity and agency and that they may not see themselves as belonging to a community of cancer survivors. Psychosocial care options: Highlights the importance of providing integrated psychosocial services for cancer survivors post-treatment, and suggests that support groups are one of the possible options. Reviews the literature on the benefits and challenges of various types of support groups, such as peer or professionally led, online or in-person, and psycho-educational or emotional.	severe cancer. They did not associate support groups with informational or instrumental support, which they valued more and sought from other informal networks or trusted physicians. Support groups are seen as a reminder of cancer when participants want to move on: Participants wanted to return to their normal lives after completing their primary treatment and starting their anti-hormonal medication. They did not want to keep cancer in their lives or face the negative emotions of other group members. They also felt that their concerns were not worthy of or suitable for support groups compared to

					those of other women with worse prognoses.
Gripsrud, B. H., Brassil, K. J., Summers, B., Søiland, H., Kronowitz, S., & Lode, K. (2016). Capturing the experience: Reflections of women with breast cancer engaged in an expressive writing intervention. *Cancer Nursing*, *39*(4), E51.	Longitudinal design: The study followed a descriptive and comparative design, collecting data from participants at two time points: baseline and one year after surgery. The aim was to explore and describe the experience and feasibility of expressive writing for women with breast cancer following mastectomy and reconstructive surgery. Expressive writing intervention: The study used a therapeutic method developed by James	The experience of expressive writing: The study aimed to explore and describe how the participants felt and thought about the writing process, what challenges and benefits they encountered, and how the writing affected their emotional and physical well-being. The experience of breast cancer, mastectomy and reconstruction: The study aimed to investigate how the participants coped with the diagnosis and treatment of breast cancer, how they perceived and related to their bodies and breasts, and how they adjusted to the changes	Women with breast cancer: The study enrolled women who had been diagnosed with breast cancer and had undergone mastectomy, either with immediate or delayed reconstructive surgery. Diverse age and ethnicity: The study included women who were aged 18 and older, and who had different ethnic and cultural backgrounds. The US sample consisted of 7 women, of whom 4 were Caucasian, 2 were African American, and 1 was Asian. The Norwegian sample consisted of 14 women, of whom 13 were Caucasian and 1 was Asian. English speakers and	Culture: explores the cross-cultural differences between American and Norwegian women with breast cancer, and how expressive writing may be influenced by their cultural backgrounds. For example, one participant from North-East Asia had a different response to the writing intervention than the other six participants, who were more comfortable with sharing their emotions and stories. Discusses how breast cancer and mastectomy can affect the meaning of breasts in different social and cultural contexts. Identity: examines how expressive	Writing as process: The participants described how they organized their writing, how much time they spent, and how they felt during and after writing. They also expressed awareness of the readership of their texts, such as the researchers, their families, or other women with breast cancer. Writing as a means to help others: The participants expressed a desire to help others through their writing, either by sharing their stories, providing support, or contributing to research. They also

	W. Pennebaker, which involves writing about one's deepest thoughts and feelings about a traumatic or stressful life event. The participants were asked to undertake four episodes of expressive writing at home, either by hand or electronically, according to an instruction tailored to breast cancer experiences (Appendix A). Semi-structured interviews: The study used a loosely structured interview guide to explore the women's experiences with the intervention (Appendix	and challenges in their psychosocial and cultural contexts. The comparison of data across countries and time points: The study aimed to conduct comparative data analyses between the US and Norway, and between baseline and one-year follow-up, to examine the similarities and differences in the participants' experiences and outcomes.	writers: The study required the participants to be able to read and write English, as the intervention and the interviews were conducted in English.	writing can help women with breast cancer to reconstruct their identities after losing a part of their body and facing a life-threatening illness. Writing can provide a way of affirming one's decisions, values, and strengths, as well as coping with the changes and challenges brought by the disease. Shows how writing can facilitate existential learning and transformation for cancer patients. Psychosocial care: Evaluates the feasibility and benefits of expressive writing as a psychosocial intervention for women with breast cancer. Writing can have positive effects on physical and emotional well-being, quality of life,	hoped that their writing would have a positive impact on the readers. Writing as therapeutic: The participants reported various benefits of writing, such as processing their emotions, validating their decisions, realizing their strengths, and coping with their challenges. They also identified some difficulties or discomforts of writing, such as recalling painful memories, confronting unpleasant feelings, or revealing intimate details. A divergent case: One participant had a different cultural background and a different response to writing. She found writing to be painful and scary, and

	B). The interviews were conducted by the researchers either in person or by phone and lasted for one to two hours. The interviews were audio-recorded and transcribed verbatim. Experientia l thematic analysis: The study used a qualitative method to analyze the interview data, which involved identifying meaningful units, generating descriptive codes, and developing themes that captured the manifest content of the participant s' experience s.			fatigue, post-traumatic stress, and negative thought patterns. Highlights the importance of timing, setting, and instruction for the implementati on of the intervention. Expressive writing can be a valuable tool for healthcare providers to introduce into the plan of care for patients with breast cancer.	she avoided thinking or talking about her illness. She also did not want to share her writing with anyone. However, she also recognized some positive aspects of writing, such as clarifying her worries and finding solutions.
Haq, R., Heus, L., Baker, N. A., Dastur,	Qualitati ve approac	The information needs of	Breast cancer patients: 39 women who	Patient-centered care: The study	BCPs and FPs had different and

D., Leung, F. H., Leung, E., ... & Parsons, J. A. (2013). Designing a multifaceted survivorship care plan to meet the information and communication needs of breast cancer patients and their family physicians: results of a qualitative pilot study. *BMC medical informatics and decision making*, *13*(1), 1-13. Canada	h to understand and address the information needs of breast cancer patients and their family physicians during the follow-up phase of care. Emergent and iterative design, with qualitative data collected before and after the development of a multi-component survivorship care plan (SCP). Three phases of data collection and analysis: 1) a needs assessment using focus groups and individual interviews	breast cancer patients, family physicians, and oncology specialist health care providers (OHCPs) regarding follow-up care after treatment. The development and implementation of a pilot SCP that consists of web-based and paper-based tools to address the identified information needs. The evaluation and feedback of the pilot SCP from the perspectives of the three stakeholder groups.	had completed or were about to complete active treatment for breast cancer at a single tertiary-care academic teaching hospital in Toronto, Canada. They had a median age at diagnosis of 55.5 years, and most had stage 1 or 2 invasive ductal carcinoma. They received various types of surgery, radiation therapy, chemotherapy, and endocrine therapy. Family physicians: 13 doctors who had some experience providing care to breast cancer patients in their respective practices. They had varying lengths of practice experience, age, and	emphasizes the importance of providing information and support that is tailored to the individual needs and preferences of breast cancer patients and their family physicians. It also recognizes that patients have different information needs at different stages of their cancer journey and that these needs are influenced by their personal and social contexts. Communication and coordination: The study identifies gaps and challenges in the communication and coordination of care between oncology specialists, family physicians, and breast cancer patients. It also explores	evolving information needs throughout the cancer journey. BCPs wanted more detailed and tailored information on a broad range of topics, while FPs wanted concise and practical information on follow-up monitoring and management. Both groups preferred web-based and paper-based formats for receiving information. The pilot SCP was perceived to be an improvement from the previous standard of care and to address many of the information needs and gaps identified by BCPs and FPs. BCPs liked the SCP website and booklet but wished they had

	with breast cancer patients, family physicians and oncology specialist health care providers; 2) a SCP design based on the findings from phase 1, consisting of web-based and paper-based tools; and 3) an evaluation and feedback using focus groups and interviews with the same stakeholder groups to assess the SCP's effectiveness and usability. Purposive sampling to recruit information-rich cases from a single tertiary-care academic		gender. Oncology specialist health care providers: 9 health professionals from different disciplines, such as surgeons, nurses, and allied health practitioners. They had experience in providing information and care to breast cancer patients and their family physicians.	how a multi-component survivorship care plan can facilitate information sharing and collaboration among these stakeholders. Survivorship and follow-up psychosocial care: The study focuses on the transition from active treatment to follow-up care, and how this can be a difficult and uncertain time for breast cancer patients. It also examines the role and responsibility of family physicians in providing follow-up care, and how they may feel ill-equipped or unsupported to do so.	access to them earlier in their care. FPs liked the SCP website and clinical care plan but suggested some improvements to make them more user-friendly and accessible. The quality of the BCP-FP relationship was seen as integral to the comfort and confidence of BCPs in receiving follow-up care from their FPs. BCPs expressed varying levels of trust and satisfaction with their FPs, depending on

	teaching hospital in Toronto.				their previous interactions and experiences. FPs expressed varying levels of competence and confidence in providing follow-up care to BCPs.
Hill-Kayser, C. E., Vachani, C. C., Hampshire, M. K., Di Lullo, G., Jacobs, L. A., & Metz, J. M. (2013). Impact of internet-based cancer survivorship care plans on health care and lifestyle behaviors. *Cancer*, *119*(21), 3854-3860.	Internet-based tool: Online tool that allows cancer survivors to create personalized SCPs based on their demographics, diagnosis, and treatments. The tool provides evidence-based or consensus-based guidelines for survivorship care, such as	Survivorship care plans focuses on the concept, design, implementation, and evaluation of SCPs, which are documents that provide cancer survivors with information and guidance on their follow-up care and health maintenance. Discusses the rationale, benefits, and challenges of SCPs, as well as the recommendations from the	Cancer survivors who used the LIVESTRONG Care Plan, an Internet-based tool for creating customized SCPs. A convenience sample of 8690 users who completed the care plan questionnaire and received a care plan document between May 2010 and January	Culture: Users of the LIVESTRONG Care Plan are mostly from the United States, Canada, Australia, and Great Britain and that they are predominantly white and college-educated. Recognizes that there may be differences in the preferences and needs of cancer survivors from different cultural backgrounds	User satisfaction: The majority of users rated the information provided by the SCP as excellent, very good, or good, and reported that it was new and useful. Most users felt more informed or empowered by the SCP and would recommend it to others. Health care communication: The SCP changed the way 61% of

	surveillance for recurrence, screening for second cancers, and management of late effects. The tool also collects anonymous data from each user and sends a follow-up survey to those who provide an email address. Follow-up survey: Voluntary survey to the users who completed the SCP and provided an email address. The survey was sent one month after the receipt of the SCP and asked about the user's satisfaction, knowledge, communication, emotions, and lifestyle changes related to	IOM and the CoC regarding their use. Internet-based intervention: Describes the LIVESTRONG Care Plan, which is an online tool that generates SCPs for cancer survivors. Explains the features, content, and sources of the tool, as well as the data collection and maintenance procedures. Compares the LIVESTRONG Care Plan with other available SCP tools and templates. Impact of SCPs on cancer survivors: Reports the results of the follow-up survey that assessed the impact of SCPs on the users of the LIVESTRONG Care Plan. User	2013. The median current age of the users was 52 years, and they were a median of 2.8 years from the time of their cancer diagnosis. Survivors of breast cancer represented 42% of users, followed by survivors of hematologic malignancies (12%), gastrointestinal cancers (11%), and genitourinary cancers (10%). Users were primarily residents of the United States (83%), followed by Canada (5%), Australia (3%), Great Britain (2%), and > 80 other countries. Of the total user group, 875 (10%) voluntarily provided an e-mail address for future contact and 298 (34%) of them	and that further studies are warranted to address the satisfaction and impact of SCPs for underrepresented minority groups and persons with less formal education. Psychosocial impact of cancer: Explores how SCPs can affect the emotional well-being of cancer survivors, as well as their communication with healthcare providers and their participation in their care. Reports that most users felt more informed or empowered by the information provided by the SCP and that only a small percentage felt confused or overwhelmed. SCPs can improve the quality of life of cancer survivors by	users participated or planned to participate in their health care, and improved communication with their health care providers for 80% of those who shared it. The SCP also increased users' knowledge of late and long-term effects and medical tests. Lifestyle changes: More than half of the users reported making or planning to make lifestyle changes as a result of the SCP, such as dietary modification and increased exercise. These changes could reduce the risk of cancer recurrence and other comorbidities.

	the SCP. Analyzed the survey responses using descriptive statistics and compared them across different subgroups of users. Randomized controlled trial	satisfaction, knowledge, communication, emotions, and lifestyle changes related to the SCPs.	completed a follow-up survey 1 month after receipt of the care plan.	increasing their knowledge of the possible long-term and late effects of cancer and its treatments, and by prompting them to make positive lifestyle changes, such as dietary modification and increased exercise. Identity: examines how SCPs can help cancer survivors to define their identity as survivors and to cope with the transition from active treatment to follow-up care.	
Juarez, G., Mayorga, L., Hurria, A., & Ferrell, B. (2013). Survivorship education for Latina breast cancer survivors: empowering survivors	Qualitative approach: Qualitative approach to evaluate the Nueva Luz educational intervention content. They conducted semi-structured interviews	Breast cancer survivorship among Latinas: Address the educational needs and quality of life issues of Latina breast cancer survivors after completing primary treatment for breast cancer. Nueva Luz intervention: Describes the	Eligibility criteria: The participants were 21 years of age or older, diagnosed with stage I, II or III breast cancer, completing primary cancer treatment, self-identified as Latina, and able to read and	Culture: Discusses how Latino cultural values and beliefs, such as personalismo, familismo, simpatia, confianza, and respect, influence the design and delivery of the Nueva Luz intervention. Emphasizes the importance of	Nueva Luz intervention: Describes a bilingual (English and Spanish) quality of life (QOL) intervention for Latina breast cancer survivors. The intervention provides tailored information and

through education. *Psicooncologia, 1 0*(1), 57. DOI: 10.5209/rev_PSIC. 2013.v10.41947 . USA	with eight Latina breast cancer survivors who received the intervention and analyzed the data using thematic analysis. Randomized controlled design: Randomized controlled design to evaluate the effectiveness of the intervention on the quality of life, uncertainty, distress, and acculturation of 52 Latina breast cancer survivors. They randomly assigned the participants to either the experimental group (N=34) or the attention	development, implementation, and evaluation of a bilingual (English and Spanish) quality-of-life intervention that is culturally congruent and tailored to the needs and preferences of Latina breast cancer survivors. The intervention consists of four weekly face-to-face individual sessions and monthly telephone support sessions that cover the physical, psychological, social and spiritual domains of quality of life.	understand English or Spanish. Socio-demographic characteristics: The average age of the participants was 52 years, most of them were born in Mexico, preferred Spanish language, had high school or less education, were not married, and were unemployed or homemakers. Most of them were diagnosed with stage I breast cancer.	providing culturally congruent and linguistically appropriate education and support for Latina breast cancer survivors. Identity: Explores how Latina breast cancer survivors self-identify as Latina and how this affects their quality of life and survivorship outcomes. Examines how the intervention helps the participants to cope with the changes in their body image, sexuality, and social roles after breast cancer treatment.<br>Psychosocial care: Describes how the Nueva Luz intervention addresses the physical, psychologic	strategies to address the physical, psychological, social and spiritual well-being of the survivors. Qualitative evaluation: Reports the qualitative evaluation of the intervention based on interviews with eight Latina breast cancer survivors who received the intervention. The evaluation focused on the content, format, cultural and linguistic appropriateness, and benefits of the intervention. Positive feedback: Positive feedback from the participants, who endorsed the intervention as relevant, helpful, empowerin

	control group (N=18) and measured the outcomes at baseline, 3 and 6 months post-intervention.			al, social, and spiritual well-being of Latina breast cancer survivors. Reports how the intervention provides tailored information, strategies, and resources to help the participants manage their symptoms, distress, uncertainty, and meaning-making after breast cancer treatment. Impact of cancer: How Latina breast cancer survivors experience worse quality of life and survivorship outcomes than other ethnic groups due to contextual factors such as low socioeconomic status, lack of health insurance, low education attainment, legal status,	g and supportive. The participants appreciated the one-on-one sessions, the bilingual printed materials, the culturally congruent approach, and the opportunity to ask questions and share their concerns. Suggestions for improvement: Discusses some suggestions for improvement from the participants, such as adding more information on nutrition, sexuality and body image.

				linguistic barriers, insufficient information, immigration status, and discrimination.	
Levesque, J. V., Gerges, M., Wu, V. S., & Girgis, A. (2020). Chinese-Australian women with breast cancer call for culturally appropriate information and improved communication with health professionals. *Cancer Reports*, *3*(2), e1218.	Qualitative study: Descriptive qualitative design to explore the experience of breast cancer for Chinese–Australian women and their coping behaviors. Semi-structured focus groups and individual interviews as data collection methods, and thematic analysis as data analysis methods. Community-based participatory research: Chinese cancer support organization, CanRevive, as a partner in the recruitment and data	The psychological impact of breast cancer diagnosis and treatment on Chinese–Australian women, such as emotional distress, fear of cancer recurrence, and uncertainty. The challenges faced by Chinese–Australian women with breast cancer, such as managing treatment side effects and obtaining psychological support The social support and coping strategies used by Chinese–Australian women with breast cancer, such as engaging with a formal support group, family,	Women of Chinese heritage who were diagnosed with breast cancer in the past 2 years and living in Australia. A total of 24 women participated, with 23 in focus groups and 1 in an individual interview The participants varied in their country of birth, language spoken at home, marital status, living situation, education, employment, time since diagnosis, number and types of treatments received	Culture: Discusses how Latino cultural values and beliefs, such as personalismo, familismo, simpatia, confianza, and respect, influence the design and delivery of the Nueva Luz intervention. Importance of providing culturally congruent and linguistically appropriate education and support for Latina breast cancer survivors. Identity: explores how Latina breast cancer survivors self-identify as Latina and how this affects their quality of life and survivorship outcomes.	Psychological impact of the diagnosis: The women reported feeling sad, anxious, uncertain, and fearful of cancer recurrence after being diagnosed with breast cancer. They also felt that their self-image and identity had changed as a result of the disease. Challenges: The women faced various challenges, such as managing the physical side effects of treatment, accessing culturally appropriate information and communication, and dealing with the emotional and social impact of cancer.

	collection process. Acknowledged the need for community consultation in developing culturally appropriate resources for the target group.	and friends, using alternative medicines, dietary changes, exercise, spirituality, and cognitive reframing.		Examines how the intervention helps the participants to cope with the changes in their body image, sexuality, and social roles after breast cancer treatment.<br>Psychosocial care: Describes how the Nueva Luz intervention addresses the physical, psychological, social, and spiritual well-being of Latina breast cancer survivors. Reports how the intervention provides tailored information, strategies, and resources to help the participants manage their symptoms, distress, uncertainty, and meaning-	They also expressed a need for psychological support but had mixed views on the value and availability of such services. Social support and coping: The women used different coping strategies to adjust to their situation, such as seeking social support from family, friends, and a Chinese-specific support group (CanRevive), using alternative medicines, dietary changes, exercise, spirituality, and cognitive strategies. However, they also encountered some difficulties in obtaining and utilizing social support, such as cultural expectations of self-sacrifice, protective buffering, and

				making after breast cancer treatment.	limited disclosure
Levine, E. G., Aviv, C., Yoo, G., Ewing, C., & Au, A. (2009). The benefits of prayer on mood and well-being of breast cancer survivors. *Supportive Care in Cancer, 17,* 295-306.	Combination of quantitative and qualitative methods to examine the use and effects of prayer among breast cancer survivors from different ethnic groups. The quantitative methods included surveys of psychological, social, and spiritual variables, while the qualitative methods involved in-depth interviews of the participants' experiences with prayer.	Focused on the use of prayer as a coping strategy for breast cancer survivors and how it was related to their mood, quality of life, social support, and spirituality. Differences in the use of prayer between ethnic groups and the themes underlying the use of prayer from the qualitative interviews. Aimed to better understand the role of prayer in the lives of breast cancer survivors from different cultural backgrounds	recruited 175 women who were no longer than 4 years posttreatment for primary breast cancer (stages 0–II) from various sites in the San Francisco Bay Area. The women were from four ethnic groups: African American, Asian/Pacific Islander, Caucasian, and Latina. They also had diverse religious backgrounds, such as Catholic, Protestant, Jewish, Buddhist, and others. Reported the demographics, health status, and stage of disease of the participants.	Ethnic and religious diversity compares the use and benefits of prayer among breast cancer survivors from four ethnic groups: African American, Asian/Pacific Islander, Caucasian, and Latina. It also examines the differences in prayer rates and types among women from different religious backgrounds, such as Catholic, Protestant, Jewish, Buddhist, and Muslim. Acknowledges that spirituality and prayer are more common among African Americans than Caucasians and that there may be variations in how prayer is	Prayer rates and correlates: 81% of the women prayed, with higher rates among African Americans and Asians/Pacific Islanders than Caucasians and Latinas. Prayer was also associated with higher benefit finding, spiritual well-being, and faith, but not with mood, quality of life, or social support. Prayer themes and functions: Identifies four themes of prayer among women: petition, comfort, praise, and difficulty in praying for self. Prayer was used to cope with cancer by asking for

				practiced and perceived among people from other ethnicities. Prayers as a coping strategy explores how prayer helps women cope with their cancer diagnosis, treatment, and survivorship. It identifies several types of prayer, such as petitionary, praise, existential, and emotional, and how they are related to mood, quality of life, social support, and benefit finding. Prayer can provide comfort, strength, guidance, courage, peace, and positive reframing for women who face the challenges of breast cancer. Prayers as a source of social support examines the role of prayer in enhancing	healing, guidance, or strength; finding peace, solace, or hope; expressing gratitude, joy, or forgiveness; and overcoming guilt, fear, or anger.

				social support for women with breast cancer. It discusses how prayer can involve a community of other worshippers, family members, friends, or spiritual leaders who pray for or with the women. It also explores how prayer can foster a sense of connection, compassion, and forgiveness with God or a higher being.	
Lopez-Class, M., Perret Gentil, M., Kreling, B., Caicedo, L., Mandelblatt, J., & Graves, K. D. (2011). Quality of life among immigrant Latina breast cancer survivors: realities of culture and enhancing cancer care. *Journal of Cancer Education,*	Data collection: The researchers collected data from 28 Latina breast cancer survivors in two ways: individual in-depth interviews and focus groups. They used structured interview guides to elicit informati	Cultural influences on quality of life: The researchers identified several themes relevant to cultural values and immigration issues that impacted the quality of life of the Latina breast cancer survivors. These themes included: relationship	Demographics: The participants were Latina breast cancer survivors who were 21 years or older, within 10 years of their diagnosis, and able to provide written informed consent in English or Spanish. They were predominantly from	Cultural values and beliefs: Explores how various cultural concepts, such as personalismo, familism, fatalism, machismo, and spirituality, influence the quality of life and survivorship experiences of Latina breast cancer survivors. Discusses how these	Relationship with God/Spirituality/World View: Most women relied on their faith and spirituality to cope with their diagnosis and treatment, although some experienced a crisis of faith or questioned their relationship with God.

26(4), 724-733.	on about general quality of life, emotional and physical impacts, family and spousal relationships, social activities, and cultural beliefs and values. They also collected demographic and clinical information from the participants. The interviews and focus groups were conducted in Spanish, audiotaped, transcribed, and translated into English. Data analysis: The researchers used NVIVO 8 software, a qualitative	with God/spirituality/world view, fatalism, personalismo, secrecy/shame surrounding breast cancer, self-reliance, machismo, familism, and factors associated with immigration. Communication with health care professionals: The researchers also reported that many participants experienced language barriers that complicated their survivorship care. They noted problems with the interpretation process, communication of their concerns and questions, understanding of medical information and treatment options, and follow-up care. They	Central and South America, with El Salvador being the most common country of origin. They were mostly monolingual in Spanish and had lived in the U.S. for an average of 10 years. They had varying levels of education, with about half having a high school degree or less. They were an average of 47 years old and 3.1 years post-diagnosis. Clinical: The participants were all diagnosed and treated in the U.S. and most of them had undergone mastectomy and chemotherapy. About half of them had received radiation	values and beliefs may differ by country of origin, length of time in the U.S., and level of acculturation. Immigration and acculturation: Examines how being an immigrant in a foreign country affects the quality of life and survivorship experiences of Latina breast cancer survivors. Considers how factors such as language barriers, social isolation, lack of familiarity with the healthcare system, and changes in social habits may pose additional challenges for these women. Communication and education: Identifies several communication issues that impact the quality of life and survivorship	Some women also said that their religious beliefs influenced their treatment decisions or views on reconstruction. Fatalism: Many women initially had fatalistic views about cancer and equated it with death, but some of them changed their views after learning more about the disease and the treatment options. Some women also expressed fears of recurrence or said they would not accept further treatment if the cancer came back. Personalismo: Several women felt isolated and lonely as immigrants and cancer survivors,

	research analysis program, to analyze the transcripts. They used a thematic content analysis approach to categorize and organize content from both the interviews and focus groups. They identified a priori and data-driven themes to describe logical relationships among the participants' responses. They revised and refined the themes using an iterative process until they reached an agreement about major coding categories. They organized the results	suggested that training of oncologists in cultural competence and utilization of trained interpreters could improve the quality of communication and care for Latina survivors.	and a small number had lumpectomy. Most of them were still on hormone therapy at the time of the study. The researchers did not collect detailed information about the stage of breast cancer at diagnosis.	experiences of Latina breast cancer survivors, such as language difficulties, use of interpreters, secrecy or shame about cancer, and lack of information about treatment options and follow-up care. Potential educational interventions to improve cancer care and outcomes for these women, such as culturally competent oncology teams, patient navigation programs, and efforts to reduce cancer fatalism. Gender roles and relationships explores how gender roles and relationships affect the quality of life and survivorship experiences of Latina breast cancer survivors, such as the impact of	and said they had fewer friends or social activities in the United States than in their home countries. Some women also said they felt less comfortable sharing their thoughts and feelings with others or reaching out for support. A few women, however, reported that their illness experience improved their friendships or social support. Secrecy/Shame Surrounding Breast Cancer: Some women did not disclose their diagnosis or emotional reactions to their family, friends, or partners, either because they felt ashamed,

	by theme and extracted exemplary quotes.			machismo on partner support and sexual functioning, the role of family as a source of support or stress, and the sense of self-reliance or independence among some women.	embarrassed, or reserved, or because they did not want to burden or worry them.
Lopez-Class, M., Gomez-Duarte, J., Graves, K., & Ashing-Giwa, K. (2012). A contextual approach to understanding breast cancer survivorship among Latinas. Psycho-Oncology, 21(2), 115-124.	Literature review: Four databases and reference lists for articles published between January 2000 and March 2010 that addressed HRQOL-related factors among Latina breast cancer survivors. They selected 37 studies that met their inclusion criteria and evaluated them based on	Health-related quality of life (HRQOL): one's subjective sense of well-being in response to a major illness. Reviews the literature on how HRQOL is measured and influenced by various factors among Latina breast cancer survivors. Social determinants of health: Ecological and contextual factors that account for significant variance in HRQOL outcomes. Discusses how socioeconom	Latina breast cancer survivors: Latina/Hispanic women who had been diagnosed with breast cancer and were either undergoing treatment or had completed treatment. The studies included in the review had different definitions of survivorship, ranging from the day of diagnosis to 5+ years post-diagnosis. Multiethnic samples: Most of the studies (n=25) included multiethnic samples that explored differences	Cultural diversity and health disparities: Heterogeneity and the rapid growth of the Latino population in the US, and the need to understand the cancer outcomes among this group. It also highlights the existing disparities in HRQOL and survivorship care among Latina breast cancer survivors compared to other ethnic groups. Contextual and ecological model: Synthesized model that incorporates multiple levels of influence	HRQOL is a complex and multidimensional construct that includes physical, functional, emotional, cognitive, and spiritual well-being. Latina breast cancer survivors face unique challenges and disparities in HRQOL compared to other ethnic groups, such as poorer psychological and emotional well-being, lower social support, higher co-morbidities, and more barriers to access and use health care

	study purpose, research design, measure of HRQOL, participant demographics, and results. Contextual and Ecological HRQOL Model: Synthesized model that combines the Contextual Model of HRQOL and the Ecological Model to examine the HRQOL literature on LABCS. The model outlines how multiple levels of influence (intrapersonal, interpersonal, institutio	ic status, cultural values, health care system, cancer-related medical factors, and public policies affect the HRQOL of Latina breast cancer survivors. Cancer survivorship and disparities: Need for research and interventions that address the specific needs and challenges of Latina breast cancer survivors. Gaps in the existing literature and the potential implications for improving health outcomes and reducing disparities in this population.	among Latina, White, Black, and Asian survivors. The studies had different methods of measuring ethnicity, such as self-identification, country of origin, or acculturation status. Diverse and heterogeneous population: The studies had different geographic locations, mostly in California, but also in the Northeast, Southeast, Southwest, and multiple states across the US. The studies also had different sample sizes, ranging from 10 to 1,837 participants. The studies reported various demographic characteristics of the participants, such as age, income, education, marital status, immigration status, and	(intrapersonal, interpersonal, community, institutional, and public policy) on the HRQOL of Latina breast cancer survivors. It argues that a comprehensive approach that considers both individual and systemic factors is required to address the unique challenges and needs of this population. Intrapersonal factors and HRQOL domains: Reviews the literature on various intrapersonal factors that affect the HRQOL of Latina breast cancer survivors, such as health status, age, psychological/ emotional well-being, cognitive well-being, functional well-being, spirituality, physical functioning,	services. Most of the existing research on HRQOL of Latina breast cancer survivors focuses on the intrapersonal and interpersonal levels, while there is a lack of studies that address the community, institutional, and public policy levels. There is a need for more longitudinal, intervention, and multi-level research that considers the contextual and ecological factors that affect HRQOL of Latina breast cancer survivors and develops culturally appropriate and tailored strategies to improve their health outcomes and reduce disparities.

	nal, community, and public policy) interact to determine HRQOL. Classified the studies according to the levels and domains of the model and identified conceptual gaps and intervention targets for LABCS.		language proficiency.	and acculturation. Interpersonal factors and social support: Discusses the role of social networks and social support in the breast cancer experience of Latinas. It identifies different sources and types of support, and how they relate to coping, decision-making, and HRQOL outcomes.	
Nápoles, A. M., Santoyo Olsson, J., Chacón, L., Stewart, A. L., Dixit, N., & Ortiz, C. (2019). Feasibility of a mobile phone app and telephone coaching survivorship care planning program among Spanish-speaking breast cancer survivors. *JMIR cancer*, *5*(2), e13543.	Mixed-methods study that combines quantitative and qualitative data to evaluate a survivorship care planning program (SCPP) for Spanish-speaking Latina breast cancer survivors. The SCPP consists of four compone	Interested in developing and testing a culturally and linguistically suitable SCPP for Spanish-speaking Latina breast cancer survivors, who face disparities and unmet needs in survivorship care. Examining the feasibility, acceptability, and preliminary efficacy of the	All participants are foreign-born and limited English proficient, most have an elementary school education or less, over half are of Mexican origin, and all have public health insurance. About half of the participants report financial hardship in the past year,	Culture: focuses on the needs and experiences of Spanish-speaking Latina breast cancer survivors, who are a culturally diverse group with different national origins, immigration histories, and acculturation levels. Cultural and linguistic barriers that these women face in accessing and understandin	The study developed and tested a multicomponent survivorship care planning program for Spanish-speaking Latina breast cancer survivors. The program included a written care plan, a survivorship booklet, a mobile app with an activity tracker, and

	nts: a written survivorship care plan (SCP), a survivorship information booklet, a mobile app with an activity tracker, and telephone coaching calls. Single-arm feasibility design, with pre- and post-intervention assessments of outcomes such as fatigue, health distress, knowledge, self-efficacy, emotional well-being, depression and somatic symptoms, and physical activity levels. Uses implementation process evaluation	SCPP, which uses a mobile app, an activity tracker, and telephone coaching to provide information, support, and motivation for managing cancer survivorship and physical activity. Exploring the potential benefits of the SCPP for improving symptoms, knowledge, self-efficacy, and well-being among the participants, as well as the barriers and facilitators for using the SCPP.	and most report a comorbid chronic condition. The majority have breast-conserving surgery and both radiation and chemotherapy and are diagnosed with invasive-ductal breast cancer. The mean age of participants is 55.8 years, and the mean time since diagnosis is 2.9 years. Only one participant reports not owning a mobile phone, and about half report using their mobile phone to access the internet.	g survivorship care information and services. Describes how the intervention was culturally and linguistically tailored to meet the preferences and needs of this population, such as using Spanish-language materials, providing health coaching by a bilingual-bicultural Latin American–trained internist, and incorporating social support and encouragement from family and friends. Identity: Explores how the intervention influenced the women's sense of self-efficacy, emotional well-being, and body image after cancer treatment. Reports that the women	health coaching calls. The study evaluated the feasibility, acceptability, and preliminary efficacy of the program using mixed methods. Feasibility and acceptability were measured by implementation processes, debriefing interviews, and satisfaction surveys. Efficacy was measured by changes in symptoms, knowledge, well-being, and physical activity. The study found preliminary evidence of program feasibility, acceptability, and efficacy, with significant 2-month improvements in fatigue, health distress, emotional well-being

	indicators, debriefing interviews, and satisfaction surveys to examine the acceptability and feasibility of the SCPP. Recruits 23 participants from two public hospitals in Northern California, using eligibility criteria such as being a Spanish-speaking Latina, having nonmetastatic breast cancer, and being within one year of termination of active treatment. Provides participants with an iPhone and an activity tracker and conducts			felt more confident in managing their health care and health after cancer, more relaxed and less stressed, and more positive about their physical appearance and weight. Women experienced a shift from being extrinsically motivated by the app and coach to being intrinsically motivated to walk and take care of themselves for their health and well-being. Psychosocial care: emphasizes the importance of providing psychosocial support and information to cancer survivors, especially those who have unmet medical, psychosocial, and informational needs.	and knowledge of follow-up care. Participants also increased their average daily steps significantly. The study also identified some challenges and limitations, such as technical issues, low literacy, poor eyesight, fear of immigration enforcement, and lack of a control group. The study suggested that future research should tailor and test the program for different subgroups of cancer survivors and address the barriers to participation and use.

	three home visits to deliver the intervention materials, collect data, and troubleshoot technical issues.				
Singh–Carlson, S., Wong, F., Martin, L., & Nguyen, S. K. A. (2013). Breast cancer survivorship and South Asian women: understanding about the follow-up care plan and perspectives and preferences for information post-treatment. *Current Oncology*, *20*(2), 63-79.	Semistructured interviews and field notes to conduct three focus group interviews with 13 participants and 11 one-to-one interviews with 24 participants in total. The interviews were conducted by the same South Asian researcher and facilitator in the language of preference of the participants. The	The impact of breast cancer treatment on South Asian women's physical and psychosocial well-being, and how it differs from other ethnic groups or the general population. The understanding of South Asian women about follow-up care and their preferences for a survivorship care plan, and how it is influenced by their age, social, and cultural factors. The meaning of being a survivor for South Asian women, and how it is	The participants were South Asian women who had been diagnosed with nonmetastatic breast cancer and who had been discharged from the BC Cancer Agency for follow-up. The participants ranged in age from 28 to 75 years, with an even distribution of six participants for each age group: <44, 45–54, 55–64, and >65 years. The participants spoke mainly Punjabi, Hindi, Urdu,	Cultural diversity and health care experiences: South Asian women's experiences of breast cancer survivorship may differ from those of other ethnic groups because of their cultural beliefs, values, and practices. Examines how language barriers, migration status, and prior healthcare experiences may affect their access to and satisfaction with healthcare	South Asian women's experiences of breast cancer survivorship: The study explored how female South Asian breast cancer survivors (SA BCSs) from different age groups and cultural backgrounds coped with the physical and psychosocial impacts of breast cancer diagnosis and treatment, and what their preferences were for a survivorship care plan (SCP). Universal and unique themes: The study

	interview s were audio-recorded and transcribe d verbatim. Data analysis: Thematic and content analysis to identify common threads and patterns in South Asian women's experience s of breast cancer after treatment. NVivo 8 software to code, store, and organize data. Constant comparativ e analysis across the age groups to identify and confirm themes and patterns. Checked and compared the accuracy and reliability of the English	related to their faith, inner strength, fate, karma, family, community, hope, and courage.	or English, or a combination of these languages. The participants were from various Indian subgroups and religions, such as Sikh, Hindu, Christian, and Muslim. The participants had varying levels of education, employment status, treatment received, and post-treatment duration.	services in Canada. Age and life stage variations: age and life stage may influence the physical and psychosoci al impacts of breast cancer and its treatment for South Asian women. Identifies some common and unique concerns for different age groups, such as reproductiv e and pregnancy issues, employme nt and financial challenges, family and spousal support, and fear of recurrence. Quiet acceptance and coping strategies: Describes a major theme of "quiet acceptance" that emerged from the	identified some themes that were common to BCSs of other ethnicities, such as fatigue, cognitive changes, fear of recurrence, and depression. It also found some themes that were unique to SA BCSs, such as quiet acceptance, karma and fate, family, and community support, and hounsla (hope and courage). These themes reflected the influence of culture, religion, and social context on the meaning and understandin g of breast cancer and survivorship for SA BCSs. Survivorship care plan preferences: The study revealed that SA BCSs wanted a generalized SCP with individualized content that

	translation and interpretation of the Punjabi, Hindi, and Urdu interviews.			narratives of South Asian women, which reflects their sense of self, faith, and inner strength in dealing with the cancer diagnosis and treatment. Discusses how South Asian women use various coping strategies, such as family and community, hope and courage, and peer support, to adapt to the survivorship phase.	addressed their specific needs and concerns after treatment. They also preferred information on depression, peer support, spiritual and language-specific resources, and practical implications of treatment side effects on their daily lives and work.
Tompkins C, Scanlon K, Scott E, Ream E, Harding S, Armes J. Survivorship care and support following treatment for breast cancer: a multi-ethnic comparative qualitative study of women's experiences. BMC Health Services Research. 2016 Dec 1;16(1):401.	Qualitative research design with in-depth interviews as the main data collection method. The researchers used Framework Analysis to analyse the data, which involved developing an analytical framework based on a priori	Explore and compare the experiences and expectations of a multi-ethnic sample of women with breast cancer regarding post-treatment care, to understand potential barriers to receiving care and inform new models of survivorship care. Focused on three key themes: emotional response on	Women who completed treatment for primary invasive breast cancer within the previous 12 months. The women were recruited from eight hospitals in England that serve large ethnic minority populations. The women were between 34 and 84 years of age (mean	Differences in communication preferences and expectations: Women from different ethnic backgrounds may have different ways of expressing their needs and concerns, and different expectations of the role and availability of healthcare professionals, especially breast care nurses. For example, some women	Unmet needs at follow-up: Women reported that their emotional, social, and informational needs were not addressed by the hospital follow-up appointments, which focused mainly on the physical aspects of recovery and detecting recurrence. Differences by

	themes, coding and indexing the transcripts, and comparing the experiences of different subgroups of women by ethnicity, age, and socio-economic position. The researchers also used quota sampling to recruit a diverse sample of women from different ethnic groups, age groups, and socioeconomic groups, as well as different types of breast cancer treatment and time since treatment completion.	transition to follow-up, challenges communicating with healthcare professionals at follow-up, and challenges finding and accessing information and support services to address unmet needs. Commonalities and differences in the experiences and expectations of women from different ethnic, socio-economic, and age groups.	54 years). The ethnic groups of the women were White British (23), Indian (7), Pakistani (9), Asian Other (1), Black Caribbean (14), Black African (7), and Other/Mixed ethnicity (5). Of the 43 women from ethnic minority groups, 28 were born abroad and 15 were UK-born. The socio-economic position of the women was measured by the National Statistics Socio-Economic Classification (NS-SEC) and grouped into higher (35), lower (22), and other (9) categories. The types of breast cancer treatment the women received were surgery, chemotherapy, and	born overseas reported language barriers and preferred verbal communication, while some Black African women expressed relief at the end of treatment and did not feel abandoned. Cultural differences in information needs and sources: Women from minority ethnic groups may have different information needs and preferences and may face challenges in finding and accessing culturally appropriate and relevant information and support services. For example, some women could not read the	ethnicity: White British women shared more about their feelings and communication challenges than Black African women, who were more likely to accept the HCPs' authority and direction. Women born overseas reported language barriers and the need for a relative to accompany them at appointments. Black African women expressed relief at the end of treatment and anxiety about follow-up appointments, while other ethnic groups felt abandoned and vulnerable. Differences by age: Older women were more likely to report positive experiences of follow-up care and less

			radiotherapy. The time since treatment completion ranged from 0 to 12 months.	written information they were given in English, some wanted information that considered their culture and religion, and some wanted signposting to people and places that could provide such information. Psychosocial impact of cancer and its treatment: Women across ethnic groups experienced physical, emotional, and social issues following treatment, such as fear of recurrence, fatigue, body image concerns, relationship issues, loss of roles and opportunities, and difficulties returning to normal life.	likely to seek additional information and support than younger women. Younger women were more dissatisfied with the lack of emotional and social support and the information gap at follow-up. Implications for new models of care: The study suggested that new models of survivorship care should be culturally appropriate, personalized, and tailored to the needs and preferences of different groups of women.

Warmoth, K., Cheung, B., You, J., Yeung, N. C., & Lu, Q. (2017). Exploring the social needs and challenges of Chinese American immigrant breast cancer survivors: a qualitative study using an expressive writing approach. *International journal of behavioral medicine*, 24, 827-835.	Expressive writing: 27 Chinese American breast cancer survivors to write three essays in Chinese at home over 3 weeks. The essays were about their thoughts and feelings regarding cancer diagnosis and treatment, the most stressful experience, and the positive aspects of cancer. Facilitate emotional disclosure and coping among the participants. Qualitative analysis: Essays in three phases. First, they used a phenomenological approach to identify meaningful units and themes	The social needs and challenges of Chinese American immigrant breast cancer survivors, such as stigma, disclosure, communication, role conflict, language barriers, access to care, and social support. The expressive writing approach is a qualitative research method to understand the breast cancer experience of Asian American women. The cultural factors that influence the health beliefs, behaviors, and quality of life of Chinese American immigrant breast cancer survivors.	The sample consists of 27 Chinese breast cancer survivors living in Southern California, who were recruited through community-based cancer organizations. The participants were mostly married, had a college education, and had low incomes. They had been living in the USA for an average of 19 years, and most of them were born in China or Taiwan. They were diagnosed with stage I, II, or III breast cancer, and underwent various treatments, such as surgery, chemotherapy, radiotherapy, and medication.	Culture: Discusses how traditional Chinese cultural values, such as Confucianism, collectivism, and harmony, influence the breast cancer experience of Chinese American women. These women tend to suppress their emotions, avoid disclosing their diagnosis, and prioritize their family roles over their own needs. Explores how acculturation, or the process of adapting to a new culture, affects the level of social support and life stress among these women. Identity: Examines how breast cancer affects the sense of identity of Chinese American women, especially about their gender roles,	Expressive writing approach: expressive writing approach to collect and analyze the essays written by 27 Chinese American immigrant breast cancer survivors. The participants wrote about their thoughts and feelings about cancer and treatment in their native language for three weeks. Phenomenological method and a line-by-line content analysis to identify themes and subthemes in the essays. Culturally unique issues: Participants experienced several social needs and challenges related to their culture, such as cancer-related

	from the essays. Second, they conducted line-by-line content analysis to categorize the sentences into themes and subthemes. Two bilingual researchers coded the essays independently. Third, they extracted cultural meanings from the themes and subthemes and formed a comprehensive story to describe the participants' experiences. Capture the social needs and challenges of the participants with cultural sensitivity.			stigma, and appearance. Women feel ashamed, tainted, or handicapped by their disease and that they struggle with fulfilling their responsibilities as daughters, mothers, and wives. Women experience changes in their body image and self-esteem after losing their hair or breasts due to treatment. Psychosocial care and challenges: Psychosocial needs and challenges of Chinese American women with breast cancer, such as emotional expression, communication, social support, and information. Women may benefit from counselling services, patient education, and family-centered interventions	stigma, goal for harmony, barriers to expressing emotions, and women's role as major caregivers. These issues reflected the influence of traditional Chinese values, such as Confucianism and collectivism, on their perception and response to cancer. Associated with identity, emotional suppression, and relational goals. Challenges experienced as immigrants: Participants faced difficulties related to being immigrants in the USA, such as language barriers, lack of insurance unfamiliarity with the healthcare system, and lack of available

				that address their cultural beliefs and barriers.	social support in a foreign country.
Wen, K. Y., Fang, C. Y., & Ma, G. X. (2014). Breast cancer experience and survivorship among Asian Americans: a systematic review. *Journal of Cancer Survivorship, 8*, 94-107.	Systematic review method to locate and analyze studies that describe the breast cancer experience, outcomes, or interventions for Asian American women in the United States. Four databases for literature published between 1995 and 2013 used a search strategy that combined terms related to breast cancer, Asian Americans, and research methods. Contextual model of health-related quality of life	breast cancer experiences and outcomes of Asian American women, and the factors that influence their HRQOL. Individual-level and system-level dimensions of the contextual model of HRQOL, such as physical health, emotional health, health efficacy, social support, cultural factors, and healthcare system factors. Highlighted the differences among Asian American subgroups and the gaps and limitations in the existing literature. Asian American women experience disrupted HRQOL following breast	The participant characteristics of the studies varied by sample size, ethnicity, location, diagnosis duration, and language. Reported that most studies included multi-ethnic samples or focused on Chinese Americans, and most studies were conducted in California. Reported that most women had early-stage breast cancer, and their diagnosis duration ranged from less than one year to more than four years. Some studies assessed and reported acculturation levels among participants and that most studies allowed participants to complete the study in	Cultural health beliefs and practices: Asian American women's cultural background influences their health behaviors, health-related quality of life (HRQOL), and coping strategies during breast cancer survivorship. For example, some women reported feeling stigmatized, ashamed, or self-sacrificing because of their cancer diagnosis. Some women also used prayer, spirituality, and traditional values as sources of support and resilience. Acculturation and language barriers examines how Asian American women's level of acculturation,	Asian American women with breast cancer have significant physical and emotional needs, and they face challenges such as language barriers, cultural beliefs, immigration stress, and discrimination in accessing health care and information. Asian American women have lower rates of choosing lumpectomy and adjuvant therapy, seeking psychosocial services, and resolving their physical issues than Caucasian women. Asian American women report different sources of

	(HRQOL) as a framework to organize and summarize the findings. 26 studies that met the inclusion criteria, which were coded and reviewed by the authors.	cancer diagnosis and treatment, and that culturally tailored, and linguistically appropriate interventions are needed to improve their survivorship	their native languages.	which is determined by factors such as place of birth, length of residency, and English proficiency, affects their breast cancer experience and outcomes. For example, some studies found that less acculturated women had lower HRQOL, more symptom distress, less social support, and more difficulty in accessing health care and information than more acculturated women. Ethnic diversity and heterogeneity and its relationship with specific needs and responses: Asian Americans are a diverse and heterogeneous group	social support, spirituality, and coping strategies than other ethnic groups, and these factors influence their HRQOL outcomes. There are variations in breast cancer experience and HRQOL within Asian American subgroups, depending on their acculturation level, place of birth, education, income, and ethnicity. There is a lack of longitudinal and intervention studies, as well as studies on Asian American subgroups, especially outside of California. More research is needed to develop culturally tailored and linguistically appropriate interventions

				with different subgroups that may have different responses and needs regarding breast cancer survivorship.	to improve HRQOL and reduce health disparities among this population

Table S4. Meta-aggregation of synthesized findings, context, codes and categories

**Findings (2)**	**Context (226)**	**Codes (113)**	**Categories (6)**
Synthesized finding 1: Lived experiences of cultural considerations in care	1. Difficulties in recruiting participants (Addington et al., 2018).<br>2. Cancer-related barriers (Addington et al., 2018).<br>3. Online yoga program, including attendance at the yoga classes (Addington et al., 2018).<br>4. Online yoga program using satisfaction ratings (Addington et al., 2018).<br>5. Improve the online yoga program (Addington et al., 2018).<br>6. Acceptability of the online yoga program (Addington et al., 2018).<br>7. Cancer-related barriers (Addington et al., 2018).<br>8. Simplifying the technology interface (Addington et al., 2018).<br>9. Minimizing burden (Addington et al., 2018).<br>10. Psychosocial challenges (Ahmed et al., 2016).<br>11. Stakeholder engagement (Ahmed et al., 2016).<br>12. Young Breast Cancer Survivors (Ahmed et al., 2016).<br>13. Intervention integrated into oncology care (Ahmed et al., 2016).<br>14. Breast cancer rehabilitation (Brennan et al., 2020).<br>15. Concept and content of survivorship care plans (Burg et al., 2009).<br>16. Minority breast cancer survivors (Burg et al., 2009).<br>17. Implementing survivorship care plans (Burg et al., 2009).<br>18. Health-related quality of life (Lopez-Class et al., 2012).<br>19. Challenges and needs of population (Lopez-Class et al., 2012).<br>20. Mobile app and an activity tracker (Lopez-Class et al., 2012).<br>21. Digital health interventions (Lopez-Class et al., 2012).<br>22. Language barriers and unfamiliarity with the system (Warmoth et al., 2017).<br>23. Need for culturally sensitive healthcare services (Warmoth et al., 2017).<br>23 statements	1. Accessibility for the program<br>2. Recruitment<br>3. Retention<br>4. Adherence<br>5. Focused feedback<br>6. Satisfaction<br>7. Qualitative feedback<br>8. Improvement<br>9. Class time<br>10. Enrolment timing<br>11. Technology interface<br>12. Participant burden<br>13. Psychosocial intervention program<br>14. Effectiveness<br>15. Intervention program<br>16. Visions for high-quality rehabilitation<br>17. Coordination of care<br>18. HRQOL<br>19. Technology and health<br>20. Healthcare systems navigation<br>20 codes	Category 1: Development of culturally responsive care models
	1. Psychosocial care among women of colour (Ashing-Giwa et al., 2004).<br>2. Socioeconomic and cultural factors (Ashing-Giwa et al., 2004).<br>3. Emotional support after breast cancer surgery (Brennan et al., 2020).	1. Psychosocial care<br>2. Unmet needs and lack of support<br>3. Barriers to psychosocial services use	Category 2: Barriers and gaps in culturally responsive care in rural communities

	4. Isolation, fear, and anxiety during rehabilitation (Brennan et al., 2020).<br>5. Barriers that hinder the use of psychosocial services (Costas-Muñiz et al., 2021).<br>6. Mental health support (Costas-Muñiz et al., 2021).<br>7. Availability of culturally and linguistically appropriate services (Costas-Muñiz et al., 2021).<br>8. Access to psychosocial services (Costas-Muñiz et al., 2021).<br>9. Mental health support (Costas-Muñiz et al., 2021).<br>10. Communication and coordination of care (Haq et al., 2013).<br>11. Information sharing and collaboration (Haq et al., 2013).<br>12. Health status, age, psychological/emotional well-being, cognitive well-being, functional well-being, spirituality, physical functioning, and acculturation (Lopez-Class et al., 2012).<br>13. Associations and implications for cultural interventions (Lopez-Class et al., 2012).<br>14. Availability of healthy foods, exercise facilities, cancer resources, insurance, language barriers, legal status, continuity of care, immigration and health care policies (Lopez-Class et al., 2012).<br>15. Access and quality of survivorship care (Lopez-Class et al., 2012).<br>15 statements	4. Unique patient, providers, and system related barriers<br>5. Insights into barriers and utilization patterns<br>6. Communication and coordination<br>7. Intrapersonal factors and HRQOL domains<br>8. Community, institutional, and public policy factors<br>8 codes	
	1. Types and sources of information about cancer diagnosis, treatment, and follow-up (Burg et al., 2009)<br>2. Information gaps, confusion, and dissatisfaction with the communication from their oncologists and primary care providers (Burg et al., 2009).<br>3. Race-related side effects and support resources (Burg et al., 2009).<br>4. Confusion and a lack of clarity regarding the transition from "active treatment" to "survivorship" (Burke et al., 2016).<br>5. Discrepancy in understanding between patients and healthcare providers (Burke et al., 2016).<br>6. Need for new terms that better capture the ongoing nature of their treatment experiences (Burke et al., 2016).<br>7. Knowledge from community organizations and support groups (Burke et al., 2016).<br>8. Lacked information from healthcare providers, contributing to a sense of uncertainty and anxiety (Burke et al., 2016).<br>9. Frequency and types of screenings they should undergo during survivorship (Burke et al., 2016).	1. Information needs<br>2. Need for information and education on the transitions to survivorship<br>3. Information needed and often not obtained from providers<br>4. Screening and monitoring<br>5. Recurrence<br>6. Patient-centered care<br>7. Communication and education<br>8. Survivorship care plan preference<br>9. Differences in communication preferences and expectations<br>10. Cultural differences in information needs and sources<br>11. Ethnicity, diversity, inclusion, Indigeneity, heterogeneity, and its	Category 3: Patient information, education and culturally responsive care

10. Fears and uncertainties about cancer recurrence, seeking guidance on recognizing symptoms and how to react (Burke et al., 2016).<br>11. Needs and preferences of breast cancer patients and their family physicians (Haq et al., 2013).<br>12. Information needs at different stages of their cancer journey, personal and social contexts (Haq et al., 2013).<br>13. Language difficulties, use of interpreters, secrecy or shame about cancer, and lack of information about treatment options and follow-up care (Lopez-Class et al., 2011).<br>14. Culturally competent oncology teams, patient navigation programs, and efforts to reduce cancer fatalism (Lopez-Class et al., 2011).<br>15. Preferences for the content and format of a survivorship care plan from active treatment to follow-up care (Singh–Carlson et al., 2013).<br>16. Information on side effects, lifestyle choices, resources, and follow-up tests (Singh–Carlson et al., 2013).<br>17. Different ways of expressing their needs and concerns, and different expectations of the role and availability of healthcare professionals (Tompkins et al., 2016).<br>18. Language barriers and preferred verbal communication, relief at the end of treatment and did not feel abandoned (Tompkins et al., 2016).<br>19. Different information needs and preferences and may face challenges in finding and accessing culturally appropriate and relevant information and support services (Tompkins et al., 2016).<br>20. Could not read the written information they were given in English, some wanted information that considered their culture and religion, and some wanted signposting to people and places that could provide such information (Tompkins et al., 2016).<br>21. Different responses and needs regarding breast cancer survivorship (Warmoth et al., 2017).<br>22. Differences in physical and emotional functioning, social network size and composition, cultural health beliefs, and doctor-patient communication (Warmoth et al., 2017).<br>22 statements	relationships with specific needs and responses<br>11 codes	
1. Culture influences the breast cancer experience of women from different ethnic groups, health beliefs, coping strategies, family support, spirituality, and access to care (Ashing-Giwa et al., 2004).<br>2. Culturally responsive health care services and programs (Ashing-Giwa et al., 2004).	1. Cultural stigma and breast cancer experiences<br>2. Unique Concerns Culture and health literacy<br>3. Cultural differences in support seeking<br>4. Cultural values	Category 4: Cultural stigma, and how it relates to self-perception of access, use and role of healthcare providers

3. Socioeconomic, cultural, and linguistic issues, impacting their access to psychosocial and mental health services (Costas-Muñiz et al., 2021).<br>4. Disparities in mental health outcomes (Costas-Muñiz et al., 2021).<br>5. Cultural competence and health literacy in promoting breast cancer prevention and screening among immigrant women (Crawford et al., 2015).<br>6. Culturally appropriate and linguistically accessible information and support to immigrant women, and how this enhances their knowledge, attitudes, and behaviours regarding breast health (Crawford et al., 2015).<br>7. Support-seeking literature suggests differences among individuals from different cultural backgrounds (Green et al., 2018).<br>8. Culture may influence how older breast cancer survivors perceive and utilize support groups (Green et al., 2018).<br>9. Cross-cultural differences and expressive writing may be influenced by their cultural backgrounds (Gripsrud et al., 2016).<br>10. Comfortable with sharing their emotions and stories (Gripsrud et al., 2016).<br>11. Affect the meaning of breasts in different social and cultural contexts (Gripsrud et al., 2016).<br>12. Survivorship care plan (Hill-Kayser et al., 2013).<br>13. Different cultural backgrounds (Hill-Kayser et al., 2013).<br>14. Personalismo, familismo, simpatia, confianza, and respeto, influence the design and delivery of the Nueva Luz intervention (Juarez et al., 2013).<br>15. Culturally congruent and linguistically appropriate education and support for Latina breast cancer survivors (Juarez et al., 2013).<br>16. Self-identify as Latina and how this affects their quality of life and survivorship outcomes (Juarez et al., 2013).<br>17. Changes in their body image, sexuality, and social roles after breast cancer treatment (Juarez et al., 2013).<br>18. Cope with breast cancer and communicate with their healthcare professionals, family, and friends (Levesque et al., 2020).<br>19. Experiences and preferences with those of Western women and other migrant groups (Levesque et al., 2020).<br>20. Influence of cultural beliefs and values, such as qi, self-sacrifice, and harmony, on their coping and communication behaviours (Levesque et al., 2020).<br>21. Use and benefits of prayer among breast cancer survivors (Levine et al., 2009).	5. Cultural care<br>6. Cultural considerations<br>7. Cultural differences in coping<br>8. Communication<br>9. Ethnicity and religious diversity<br>10. Cultural identity, values, and beliefs<br>11. Cultural diversity and health disparities<br>12. Culturally sensitive care<br>13. Culture and language<br>14. Cross cultural communication<br>15. Health disparities due to culture<br>16. Cultural diversity and health care experiences<br>17. Culturally sensitive care<br>18. Cultural health beliefs and practices<br>19. Cultural identity, power, privilege, and positionality<br>20. Culturally appropriate care<br>20 codes

22. Differences in prayer rates and types among women from different religious backgrounds (Levine et al., 2009).
23. Spirituality and prayer are more common among African Americans than Caucasians (Levine et al., 2009).
24. Cultural concepts, such as personalismo, familism, fatalism, machismo, and spirituality, influence the quality of life and survivorship experiences of Latina breast cancer survivors (Lopez-Class et al., 2011).
25. Values and beliefs may differ by country of origin, length of time in the U.S., and level of acculturation (Lopez-Class et al., 2011).
26. Understand the cancer outcomes (Lopez-Class et al., 2012).
27. Existing disparities in HRQOL and survivorship care (Lopez-Class et al., 2012).
28. Needs and experiences of Spanish-speaking Latina breast cancer survivors, who are a culturally diverse group (Nápoles et al., 2019).
29. Cultural and linguistic barriers that these women face in accessing and understanding survivorship care information and services (Nápoles et al., 2019).
30. Culturally and linguistically tailored to meet the preferences and needs of this population, social support and encouragement from family and friends (Nápoles et al., 2019).
31. Lower rates of follow-up care, higher symptom burden, and poorer quality of life compared to their white counterparts (Nápoles et al., 2019).
32. Culturally and linguistically tailored interventions to reduce these disparities (Nápoles et al., 2019).
33. Cultural beliefs, values, and practices (Singh–Carlson et al., 2013).
34. Language barriers, migration status, and prior healthcare experiences may affect their access to and satisfaction with healthcare services in Canada (Singh–Carlson et al., 2013).
35. Traditional Chinese cultural values, such as Confucianism, collectivism, and harmony, influence the breast cancer experience (Warmoth et al., 2017).
36. Suppress their emotions, avoid disclosing their diagnosis, and prioritize their family roles over their own needs (Warmoth et al., 2017).
37. Acculturation, or the process of adapting to a new culture, affects the level of social support and life stress among these women (Warmoth et al., 2017).

38. Sense of identity of Chinese American women, especially in relation to their gender roles, stigma, and appearance (Warmoth et al., 2017).<br>39. Feel ashamed, tainted, or handicapped by their disease, and that they struggle with fulfilling their responsibilities as daughters, mothers, and wives (Warmoth et al., 2017).<br>40. Changes in their body image and self-esteem after losing their hair or breasts due to treatment (Warmoth et al., 2017).<br>41. Asian American women's cultural background influences their health behaviors, health-related quality of lifeand coping strategies during breast cancer survivorship (Warmoth et al., 2017).<br>42. Feeling stigmatized, ashamed, or self-sacrificing because of their cancer diagnosis (Warmoth et al., 2017).<br>43. Prayer, spirituality, and traditional values as sources of support and resilience (Warmoth et al., 2017).<br>43 statements		
1. Awareness of the impact of surgery, their expectations and reality of physiotherapy care, their confidence and challenges in doing home exercises, and their sources of information and support (Brennan et al., 2020).<br>2. Worsened mental health, and its impact on income, particularly emphasizing the challenges faced by Latinos in comparison to non-Latino Whites (Costas-Muñiz et al., 2021).<br>3. Support needs changed throughout their cancer journey, and how they faced new informational and emotional challenges in the post-treatment phase (Costas-Muñiz et al., 2021).<br>4. Existing support systems, such as family, friends, and other survivors, were not always adequate or available to meet their needs, and how they felt alone or guilty about their cancer experience (Costas-Muñiz et al., 2021).<br>5. Support groups could potentially address gaps and provide consistent and accurate information, validation, and mutual help (Green et al., 2018).<br>6. Impact of cancer on various aspects of women's lives, such as their relationships, work, self-image, and outlook (Gripsrud et al., 2016).<br>7. Writing can help women to process and articulate their cancer experiences, and to find meaning and hope in their stories (Gripsrud et al., 2016).<br>8. Emotional and cognitive challenges that women face when confronting their illness reality, and how writing can help them to manage their worries and emotions (Gripsrud et al., 2016).<br>9. Latina breast cancer survivors experience worse quality of life and survivorship outcomes than other	1. Rehabilitation experiences<br>2. Financial impact of cancer diagnosis<br>3. Impact of cancer on support needs<br>4. Impact of cancer on cultural care<br>5. Impact of cancer on family and children<br>6. Impact of cancer on young adolescents and adults<br>7. Psychosocial impact of cancer and its treatment<br>8. Impact of cancer on wellbeing<br>8 codes	Category 5: Impact of cancer and linguistically appropriate care

	ethnic groups due to contextual factors such as low socioeconomic status, lack of health insurance, low education attainment, legal status, linguistic barriers, insufficient information, immigration status, and discrimination (Juarez et al., 2013).<br>10. Quality of life and survivorship outcomes of Latina breast cancer survivors by addressing their unmet needs and concerns (Juarez et al., 2013).<br>11. Impact of cancer and its treatment on the women's physical, emotional, and social functioning (Nápoles et al., 2019).<br>12. Women experienced symptoms such as pain, swelling, constipation, sleep problems, and depression (Nápoles et al., 2019).<br>13. Women faced challenges such as financial hardship, low literacy, limited English proficiency, and lack of access to quality health care (Nápoles et al., 2019).<br>14. Cope with these challenges and improve their quality of life (Nápoles et al., 2019).<br>15. Physical, emotional, and social issues following treatment, such as fear of recurrence, fatigue, body image concerns, relationship issues, loss of roles and opportunities, and difficulties returning to normal life (Tompkins et al., 2016).<br>16. Felt unprepared and unsupported for life after treatment, and that their emotional and social needs were overlooked or unmet by the traditional model of follow-up care (Tompkins et al., 2016).<br>17. Impact of breast cancer on the quality of life and well-being of Chinese American women (Tompkins et al., 2016).<br>18. Negative emotions, such as fear, anger, depression, and distress, as well as physical symptoms, such as pain, fatigue, and nausea (Warmoth et al., 2017).<br>19. Multiple sources of stress, such as language barriers, lack of insurance, unfamiliarity with the healthcare system, and lack of available social support in a foreign country (Warmoth et al., 2017).<br>19 statements		
Synthesized finding 2: Lived experiences of psychosocial well-being of immigrants	1. Psychosocial issues such as anxiety, fear of recurrence, decision-making difficulties, and sexuality/relationship issues (Ahmed et al., 2016).<br>2. Psychosocial impact of breast cancer on women's quality of life, emotional well-being, and mental health (Ashing-Giwa et al., 2004).<br>3. Psychological issues such as fear, anxiety, denial, and depression (Ashing-Giwa et al., 2004).<br>4. Social resources such as family, friends, support groups, and health professionals to deal with breast cancer (Ashing-Giwa et al., 2004).	1. Psychosocial challenges of young breast cancer survivors<br>2. Psychosocial functioning and social support networks<br>3. Psychosocial, emotional well-being of breast cancer survivorship<br>4. Psychosocial impact of cancer<br>4 codes	Category 6: Challenges with psychosocial well-being and culturally responsive care

5. Psychosocial and emotional needs of breast cancer survivors, especially after the completion of active treatment (Burg et al., 2009).<br>6. Types of distress, anxiety, and uncertainty that minority breast cancer survivors experience, and the lack of adequate support and guidance from their health care providers (Burg et al., 2009).<br>7. Value of community-based, population-specific cancer support programs, such as the Sisters Network, as referral sources for primary care providers and as sources of information and empowerment for survivors (Burg et al., 2009).<br>8. Emotional well-being of cancer survivors, as well as their communication with health care providers and their participation in their care (Hill-Kayser et al., 2013).<br>9. Users felt more informed or empowered by the information, and only a small percentage felt confused or overwhelmed (Hill-Kayser et al., 2013).<br>10. Quality of life of cancer survivors by increasing their knowledge of the possible long-term and late effects of cancer and its treatments, and by prompting them to make positive lifestyle changes, such as dietary modification and increased exercise (Hill-Kayser et al., 2013).<br>10 statements		
1. Motivation, proactivity, and resilience in pursuing their recovery (Brennan et al., 2020).<br>2. Benefits of exercise, peer support, and digital technology for enhancing physical and psychological well-being (Brennan et al., 2020).<br>3. Information on self-care strategies related to diet, nutrition, exercise, and smoking cessation (Burke et al., 2016).<br>4. Clear communication, personalized information, and ongoing support for breast cancer survivors, especially during the transition from active treatment to survivorship (Brennan et al., 2020).<br>5. Provision and the potential benefits of well-designed survivorship care plans tailored to the diverse needs of breast cancer survivors (Burke et al., 2016).<br>6. Self-management strategies that Chinese-Australian women use to adjust to their breast cancer experience, such as dietary changes, exercise, spirituality, cognitive reframing, and alternative medicines (Levesque et al., 2020).<br>7. Cultural awareness and sensitivity when developing or adapting self-management resources for this minority group (Levesque et al., 2020).<br>8. Prayer helps women cope with their cancer diagnosis, treatment, and survivorship (Levine et al., 2009).	1. Self-driven rehabilitation<br>2. Self-care strategies<br>3. Self-management strategies of Chinese-Australian women<br>4. Prayer as a coping strategy<br>5. Culture and language as coping<br>6. Prayers as a personal and subjective experience<br>7. Quiet acceptance and coping strategies<br>8. Personal coping mechanism<br>8 codes	Category 4: Cultural stigma, and self-perception of access, use and role of health care providers

	9. Types of prayer, such as petitionary, praise, existential, and emotional, and how they are related to mood, quality of life, social support, and benefit finding (Levine et al., 2009).<br>10. Prayer can provide comfort, strength, guidance, courage, peace, and positive reframing for women who face the challenges of breast cancer (Levine et al., 2009).<br>11. Prayer is a personal and subjective experience that may vary depending on the individual, the situation, and the context (Levine et al., 2009).<br>12. Diversity and complexity of prayer among breast cancer survivors (Levine et al., 2009).<br>13. Prayer and its effects on health and well-being (Levine et al., 2009).<br>14. "Quiet acceptance" emerged from the narratives of South Asian women, which reflects their sense of self, faith, and inner strength in dealing with cancer diagnosis and treatment (Singh–Carlson et al., 2013).<br>15. South Asian women use various coping strategies, such as family and community, hope and courage, and peer support, to adapt to the survivorship phase (Singh–Carlson et al., 2013).<br>16. Coping mechanisms employed by the participants, such as seeking social support, using traditional Chinese medicine, and practicing religious or spiritual activities (Warmoth et al., 2017).<br>17. Coping strategies are influenced by their cultural beliefs and values (Warmoth et al., 2017).<br>17 statements		
	1. Sense of identity, such as their roles as mothers, wives, and caregivers (Ashing-Giwa et al., 2004).<br>2. Identity is shaped by their ethnicity, acculturation, socioeconomic status, and education (Ashing-Giwa et al., 2004).<br>3. Identity changes and challenges due to breast cancer (Ashing-Giwa et al., 2004).<br>4. Immigrant women from different cultural backgrounds adapt to their new host country and how they perceive their health and well-being (Crawford et al., 2015).<br>5. Peer health educator program helps immigrant women develop a sense of identity and belonging (Crawford et al., 2015).<br>6. Participants resisted an identity as a cancer patient, and how they wanted to move on from the cancer experience and return to their life before cancer (Green et al., 2018).<br>7. Attending a support group may challenge their sense of identity and agency, and they may not see themselves as belonging to a community of cancer survivors (Green et al., 2018).	1. Cultural identity and role change<br>2. Cultural identity and acculturation<br>3. Cultural identity as a cancer survivor<br>4. Cultural identity, and power<br>5. Cultural identity and privilege<br>6. Cultural immigration and acculturation<br>7. Cultural identity and positionality<br>8. Cultural changes and coping strategies<br>9. Acculturation and language barrier<br>9 codes	Category 4: Cultural stigma, and self-perception of access, use and role of health care providers

8. Cancer survivorship identity is perceived differently among women 70 years and older (Green et al., 2018).
9. Expressive writing can help women with breast cancer reconstruct their identities after losing a part of their body and facing a life-threatening illness (Gripsrud et al., 2016).
10. Writing can provide a way of affirming one's decisions, values, and strengths, as well as coping with the changes and challenges brought by the disease (Gripsrud et al., 2016).
11. Writing can facilitate existential learning and transformation for cancer patients (Gripsrud et al., 2016).
12. Define their identity as survivors and cope with the transition from active treatment to follow-up care (Hill-Kayser et al., 2013).
13. Comprehensive and customized descriptions of the needs of cancer survivors, and to address the social, economic, and emotional impacts of a cancer diagnosis (Hill-Kayser et al., 2013).
14. Empower cancer survivors to take more control of their care and to be more active and informed participants in their health care (Hill-Kayser et al., 2013).
15. Being an immigrant in a foreign country affects the quality of life and survivorship experiences of Latina breast cancer survivors (Lopez-Class et al., 2011).
16. Language barriers, social isolation, lack of familiarity with the health care system, and changes in social habits may pose additional challenges for these women (Lopez-Class et al., 2011).
17. Sense of self-efficacy, emotional well-being, and body image after cancer treatment (Nápoles et al., 2019).
18. More confident in managing their health care and health after cancer, more relaxed and less stressed, and more positive about their physical appearance and weight (Nápoles et al., 2019).
19. Shift from being extrinsically motivated by the app and coach to being intrinsically motivated to walk and take care of themselves for their health and well-being (Nápoles et al., 2019).
20. Identity changes and use different coping strategies because of their cancer experience (Nápoles et al., 2019).
21. Permanently changed by cancer and struggled to adjust to their new identity, while some wanted to move on and forget about cancer (Nápoles et al., 2019).
22. Support from other women who had breast cancer, while some avoided such contact due to fear or stigma. Some women were active help seekers, while

	some were reluctant to contact healthcare professionals or use support services (Tompkins et al., 2016).<br>23. Asian American women's level of acculturation, which is determined by factors such as place of birth, length of residency, and English proficiency, affects their breast cancer experience and outcomes (Warmoth et al., 2017).<br>24. Less acculturated women had lower health related quality of life, more symptom distress, less social support, and more difficulty in accessing health care and information than more acculturated women (Warmoth et al., 2017).<br>24 statements		
	1. Disparities in psychosocial outcomes, specifically examining how Latina individuals living with breast cancer experience poorer quality of life and higher rates of depression compared to non-Latino Whites (Costas-Muñiz et al., 2021).<br>2. Access to psychosocial services among breast cancer survivors (Costas-Muñiz et al., 2021).<br>3. Utilization rates of psychological, psychiatric, and other psychosocial interventions, with a particular focus on the differences between Latina and non-Latina White individuals (Costas-Muñiz et al., 2021).<br>4. Disparities in the use of psychosocial services, revealing that Latinos are less likely to receive these services than non-Latino Whites (Costas-Muñiz et al., 2021).<br>5. Gap, encompassing patient-, provider-, and system-related barriers (Costas-Muñiz et al., 2021).<br>6. Role of peer health educators and social networks in providing psychosocial care and empowerment to immigrant women (Costas-Muñiz et al., 2021).<br>7. Peer health educator program fosters a caring and trusting environment, where immigrant women can share their experiences, feelings, and concerns, and receive emotional and practical support (Crawford et al., 2015).<br>8. Peer health educator program empowers immigrant women to take charge of their health and to access healthcare services (Crawford et al., 2015).<br>9. Integrated psychosocial services for cancer survivors post-treatment and suggests that support groups are one of the viable options (Green et al., 2018).<br>10. Support groups, such as peer or professionally led, online or in-person, and psycho-educational or emotional (Green et al., 2018).<br>11. Support groups tailored to the needs and preferences of older breast cancer survivors, and clinicians should play a key role in promoting and	1. Disparities in psychosocial outcomes<br>2. Utilization of psychosocial Services<br>3. Psychosocial service use disparities<br>4. Psychosocial care and empowerment<br>5. Psychosocial care options/strategies<br>6. Psychosocial health and well-being<br>7. Psychosocial care<br>8. Survivorship and follow-up of psychosocial care<br>9. Psychosocial impact and needs of Chinese-Australian women<br>10. Prayers as a source of social support<br>11. Interpersonal factors and social support<br>12. Contextual psychosocial care aspects<br>13. Social support networks<br>14. Social support systems<br>15. Psychosocial care and challenges<br>16. Psychosocial interventions and resources<br>16 codes	Category 6: Challenges with psychosocial well-being and culturally responsive care

referring them to appropriate resources (Green et al., 2018).

12. Benefits of expressive writing as a psychosocial intervention for women with breast cancer (Gripsrud et al., 2016).
13. Physical and emotional well-being, quality of life, fatigue, post-traumatic stress, and negative thought patterns (Gripsrud et al., 2016).
14. Timing, setting, and instruction for the implementation of the intervention (Gripsrud et al., 2016).
15. Expressive writing in plan of care for patients with breast cancer (Gripsrud et al., 2016).
16. Transition from active treatment to follow-up care, and how this can be a difficult and uncertain time for breast cancer patients (Haq et al., 2013).
17. Role and responsibility of family physicians in providing follow-up care, and how they may feel ill-equipped or unsupported to do so (Haq et al., 2013).
18. Nueva Luz intervention addresses the physical, psychological, social, and spiritual well-being of Latina breast cancer survivors (Juarez et al., 2013).
19. Tailored information, strategies, and resources to help the participants manage their symptoms, distress, uncertainty, and meaning making after breast cancer treatment (Juarez et al., 2013).
20. Psychological impact of breast cancer diagnosis and treatment on Chinese-Australian women, such as depression, anxiety, distress, and fear of recurrence (Levesque et al., 2020).
21. Psychological needs and challenges of these women, such as the need for information, support, and culturally appropriate services (Levesque et al., 2020).
22. Self-identified need for psychological support and the availability and acceptability of such support (Levesque et al., 2020).
23. Role of prayer in enhancing social support for women with breast cancer. It discusses how prayer can involve a community of other worshippers, family members, friends, or spiritual leaders who pray for or with the women (Levine et al., 2009).
24. Prayer can foster a sense of connection, compassion, and forgiveness with God or a higher being (Levine et al., 2009).
25. Difficulty praying for themselves and may rely on others to pray for them (Levine et al., 2009).
26. Role of social networks and social support in the breast cancer experience of Latinas (Lopez-Class et al., 2012).
27. Sources and types of support, and how they relate to coping, decision-making, and HRQOL outcomes (Lopez-Class et al., 2012).

	28. Lack of social support among some Latina immigrant breast cancer survivors, and the potential benefits of peer support interventions (Lopez-Class et al., 2012).<br>29. Psychosocial support and information to cancer survivors, especially those who have unmet medical, psychosocial, and informational needs (Nápoles et al., 2019)<br>30. Written survivorship care plan, a survivorship information booklet, a mobile app with an integrated activity tracker, and health coaching calls (Nápoles et al., 2019).<br>31. Emotional well-being, reduced their fatigue and health distress and increased their knowledge of recommended follow-up care and resources (Nápoles et al., 2019).<br>32. Psychosocial needs and challenges of Chinese American women with breast cancer, such as emotional expression, communication, social support, and information (Nápoles et al., 2019).<br>33. Role of family and friends in supporting the participants' use of the intervention and their cancer survivorship journey (Nápoles et al., 2019).<br>34. Social support in health behaviour changes and cancer survivorship (Nápoles et al., 2019).<br>35. Women may benefit from counselling services, patient education, and family-centred interventions that address their cultural beliefs and barriers (Warmoth et al., 2017).<br>36. Services in native language and by culturally competent professionals (Warmoth et al., 2017).<br>37. Culturally relevant and linguistically appropriate psychosocial interventions and resources for Asian American breast cancer survivors (Warmoth et al., 2017).<br>38. Asian American women expressed a desire for support groups, information on stress management and relaxation, and alternative therapies, but they also faced barriers such as limited availability, accessibility, and acceptability of these services (Warmoth et al., 2017).<br>39. Cultural and socio-ecological factors that influence Asian American women's breast cancer survivorship (Warmoth et al., 2017).<br>39 statements		
	1. Healthy eating, physical activity, and managing the effects of cancer on their daily lives (Burke et al., 2016)<br>2. Survivorship Care Plans to address specific concerns and information gaps (Burke et al., 2016).<br>3. Equip them with questions to ask their doctors and include referrals to primary care providers knowledgeable about breast cancer (Burke et al., 2016).	1. Healthy lifestyle choices<br>2. Perspectives on SCP content and delivery<br>3. Questions for doctors<br>4. Hereditary aspects<br>5. Gender roles and relationships<br>6. Empowerment	Category 6: Challenges with psychosocial wellbeing and culturally responsive care

4. Information on hereditary breast cancer and guidance on how to talk to family members about their experiences (Burke et al., 2016).<br>5. Gender roles and relationships affect the quality of life and survivorship experiences of Latina breast cancer survivors, such as the impact of machismo on partner support and sexual functioning, the role of family as a source of support or stress, and the sense of self-reliance or independence among some women (Lopez-Class et al., 2011).<br>6. Breast cancer may change women's perceptions of themselves and their roles in society (Lopez-Class et al., 2011).<br>7. Enhancing knowledge and self-efficacy in managing their health after cancer (Nápoles et al., 2019).<br>8. Empowerment in cancer survivorship care (Nápoles et al., 2019).<br>9. Challenges such as financial hardship, low literacy, limited English proficiency, and lack of access to quality health care, the participants were able to engage with the intervention and improve their health outcomes (Nápoles et al., 2019).<br>10. Resilience of the participants in overcoming adversity (Nápoles et al., 2019).<br>11. Aging, ageism, and life stage may influence the physical and psychosocial impacts of breast cancer and its treatment for South Asian women (Singh–Carlson et al., 2013).<br>12. Concerns for different age groups, such as reproductive and pregnancy issues, employment and financial challenges, family and spousal support, and fear of recurrence (Singh–Carlson et al., 2013).<br>13. Resilience and ability to find positive meaning in their cancer experience (Warmoth et al., 2017).<br>14. Post-traumatic growth refers to the positive psychological change experienced because of the struggle with highly challenging life circumstances (Warmoth et al., 2017).<br>14 statements	7. Resilience<br>8. Age and life stage variations<br>9. Resilience and growth<br>9 codes
